# Temporal Changes in the Role of Species Sorting and Evolution Determine Community Dynamics

**DOI:** 10.1111/ele.70033

**Published:** 2024-12-31

**Authors:** Julius Hoffmann, Shane Hogle, Teppo Hiltunen, Lutz Becks

**Affiliations:** ^1^ Aquatic Ecology and Evolution University of Konstanz Konstanz Germany; ^2^ Department of Biology University of Turku Turku Finland

**Keywords:** coevolution, community ecology, eco‐evolutionary dynamics, experimental evolution, genomics, microbiology, molecular evolution, predator–prey interactions, *Tetrahymena*

## Abstract

Evolutionary change within community members and shifts in species composition via species sorting contribute to community and trait dynamics. However, we do not understand when and how both processes contribute to community dynamics. Here, we estimated the contributions of species sorting and evolution over time (60 days) in bacterial communities of 24 species under selection by a ciliate predator. We found that species sorting contributed to increased community carrying capacity, while evolution contributed to decreased anti‐predator defences. The relative roles of both processes changed over time, and our analysis indicates that if initial trait variation was in the direction of selection, species sorting prevailed, otherwise evolution drove phenotypic change. Furthermore, community composition, population densities and genomic evolution were affected by phenotypic match–mismatch combinations of predator and prey evolutionary history. Overall, our findings help to integrate when and how ecological and evolutionary processes structure communities.

## Graphical abstract

Evolutionary change within community members and shifts in species composition contribute to community and trait dynamics. To integrate when and how both processes structure communities, we estimated the contributions of species sorting and evolution over time (60 days) in bacterial communities of 24 species under selection by a ciliate predator. Species sorting led to an increase in community carrying capacity, whereas evolution contributed to a decrease in anti‐predator defences, and our analysis indicates that if initial trait variation was in the direction of selection, species sorting prevailed, otherwise evolution drove phenotypic change.

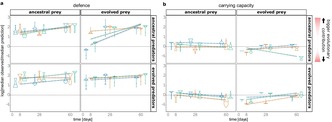

## 1 Introduction

Evolution can impact communities to a similar extent as ecology, (Bassar et al. 2010; Hairston et al. 2005; Palkovacs et al. 2009) making consideration of evolution often necessary to accurately describe community dynamics (Ellner, Geber, and Hairston 2011; Leibold et al. 2022; Schoener 2011; Thompson 1998; Toju et al. 2017), because species sorting and evolution can simultaneously affect the compositional, phenotypical and genetic make‐up of communities (Whitham et al. 2006). For example, previous adaptation of the zooplankter *Daphnia magna* to environmental conditions altered its population's phenotypical composition, which changed the outcome of species sorting in a zooplankton community (Pantel, Duvivier, and Meester 2015). Additionally, evolution in a species can alter community composition by modifying interspecific interactions (Gómez et al. 2016; Hiltunen et al. 2017; Hogle et al. 2022; Padfield et al. 2020). Changes in community composition can modify evolutionary outcomes (Friman et al. 2016; Turcotte, Corrin, and Johnson 2012), for example, through changes in community composition modifying selection (Stinchcombe and Rausher 2002; terHorst 2010), eventually diminishing evolutionary responses to selection when already well‐adapted species are favoured (Barraclough 2015). Despite these insights, our understanding of interactions between ecological and evolutionary processes and how they together shape community dynamics remains limited (Govaert et al. 2021; Yamamichi, Ellner, and Hairston 2023). Few studies have disentangled the contributions of evolution and species sorting over time to key phenotypic traits in a community (Brans et al. 2017; Jewell and Bell 2023).

Predation is a common and strong driver of selection, affecting both ecological and evolutionary dynamics of prey populations and communities. Predation on prey communities alters, for example, community composition (Burian et al. 2022; Chase et al. 2009; van Valen 1974) by facilitating coexistence if predation sufficiently differentiates between species to counterbalance differences in competitive strength (Chesson and Kuang 2008; Fairweather 1985). Additionally, prey species might cooperate by expressing a shared defence against predators (Jousset 2012), potentially allowing weakly defended species to persist. Prey often adapt rapidly by evolving anti‐predator defences, which is well‐documented in single‐species systems and individual species within communities (Abrams 2000; Jousset 2012; Matz and Kjelleberg 2005; Meyer and Kassen 2007; Yoshida et al. 2003). However, such adaptations at the community level are less studied though shifts in the phenotypic expression of microbial communities in response to predation have been described (Hahn and Höfle 1999, 2001; Mathisen et al. 2016). Prey defence evolution primarily depends on the predator's traits to overcome these defences, which evolve in response to prey defence (Hogle et al. 2023; Huang et al. 2017; Nair et al. 2019; Scheuerl et al. 2019), eventually leading to continuous predator–prey coevolution. This coevolutionary history can influence evolutionary and ecological outcomes in communities (Faillace and Morin 2020; Leibold et al. 2022). For example, shared evolutionary history of interacting species may reduce competition and facilitate coexistence by reducing niche overlap between prey (Zee and Fukami 2018).

Coevolution typically results in a phenotypic match–mismatch pattern due to inevitable delays in counter‐adaptations in interacting partners. For example, recent prey defence evolution can reduce predator consumption, while predator counter‐adaptation leads to increased consumption, impacting selection and prey and predator population sizes (Friman et al. 2008; Nair et al. 2019). Consequently, evolutionary histories may impact communities in complex ways, altering community composition, productivity, and stability. While phenotypic mismatches between predator and prey occur in nature (Hanifin, Brodie, and Brodie 2008), few studies have tested them experimentally in predator–prey communities (Hogle et al. 2023) (but see (Hiltunen and Becks 2014; Scheuerl et al. 2019) for single species systems). Disentangling the relative roles of evolutionary changes and species sorting following such match–mismatches could provide important insights into eco‐evolutionary dynamics in communities.

**FIGURE 1:**
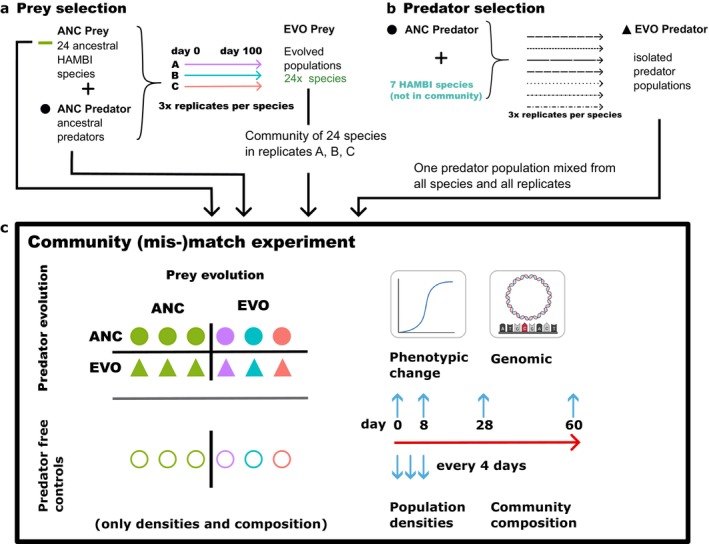
Experimental design. (a) Prey evolutionary histories: *Ancestral prey* communities (ANC) were assembled from clonal bacterial species (light green) derived from a culture collection (Microbial Domain Biological Resource Centre HAMBI, University of Helsinki, Finland). For communities with *evolved prey* (EVO), the same species were cultured separately in triplicates (A, B, C) with ancestral populations of their predator *Tetrahymena thermophila* for ~165 generations before the (mis‐)match experiment. Evolved prey from the separate replicates were then combined into three evolved communities (A, B, C). (b) Predator evolutionary histories: *Ancestral predators* (ANC) were clonal isolates of *Tetrahymena thermophila* strain 1630/1 U (CCAP) that had been maintained without bacteria; serial propagation under conditions restricted to asexual reproduction. *Evolved predators* (EVO) were pooled at equal densities from *Tetrahymena thermophila* populations derived from a previous long‐term selection: Ancestral *T. thermophila* populations were co‐cultured with each one of seven ancestral bacterial species for each species in three replicate lines; six of seven species were not part of our 24‐species community (Cairns et al. 2020). (c) Community (mis‐)match experiment: Prey and predator evolutionary histories were combined in a fully factorial design, and each combination was replicated in three microcosms. Additionally, three microcosms per prey evolutionary history were inoculated without predators (controls). The communities were propagated for 60 days as semicontinuous cultures with 30% transfer every 4 days.

Here, we studied the contributions of species sorting and evolution in shaping bacterial community dynamics under predation. For this, we co‐evolved 12 prey communities from a collection of 24 bacterial species with the protist predator *Tetrahymena thermophila* for 60 days. We studied the impact of phenotypic match–mismatch between prey communities and the predator by combining two distinct (co‐)evolutionary histories in laboratory microcosms using a fully factorial design (hereafter: (mis‐)match experiment, Figure 1). Half of the bacterial communities were composed of clonal species that grew without predator exposure prior to the experiment (hereafter, ancestral prey), and the other half was composed of populations of the same species harvested after co‐culture with *Tetrahymena* (hereafter, evolved prey). These two distinct prey histories were combined with predators that had no prior bacterial exposure (hereafter, ancestral predator) or with predators isolated from long‐term bacterial co‐cultures (hereafter, evolved predator). We treated rapid shifts in community composition (species sorting) and population densities as indicators of ecological change while using shifts in community functional traits (carrying capacity, anti‐predator defence) and bacterial non‐synonymous mutational frequencies as evolutionary change.

## 2 Materials and Methods

Full descriptions of experiments and analyses are in the Supporting Information (SI).

### 2.1 Mismatch Experiment

Prey communities and predator populations from both evolutionary histories were combined in a fully factorial design, including microcosms without predators (see Supporting Iinformation for details; Figure 1). For this, ancestral and evolved bacterial populations were revived from glycerol stocks, grown in fresh 5% King's B (KB) medium for 48 h, and adjusted to an OD 1. Three replicates for each prey history and predator condition (ancestral, evolved and no predators) were established in 6 mL of 5% KB medium. Cultures were maintained with 30% transfer every 4 days for 60 days (14 transfers), with samples taken at each transfer (SI). Predators were counted manually, and prey densities were estimated via OD at 600 nm.

### 2.2 Phenotypic Characterisation

The prey traits of carrying capacity and defence were measured following a period of isolated growth to correct for potential phenotypic plasticity and expose evolutionary changes (Wiser and Lenski 2015); bacterial stocks (initial and clonal stocks isolated from Days 8, 28 and 60 from the experimental communities) were revived in 5% KB medium, acclimatised for 24 h and replicated into fresh KB medium. Their growth was followed in the absence and presence of ancestral predators by measuring optical density (OD_600_) over 96 h. Carrying capacity was defined as OD in the absence of predators after 96 h, defence as the logarithmic ratio between OD in the presence and absence of predators and community carrying capacity and defence as the median values of the 24 clones per sample and day. A small subset of clones had higher OD values with predators and was excluded from subsequent analysis.

### 2.3 Community Composition

Community composition was analysed by performing Principal Component Analysis (PCA) on the centred log ratio transform of species' relative abundance (Aitchison distance) derived from 16 s amplicon sequencing as described in detail earlier (Hogle et al. 2022, 2023). All microcosms and sampling days (starting communities, Days 12, 28, 44 and 60 for microcosms without predator, every 4 days with predator) were included, and predator density and time were projected as environmental variables. We then geometrically analysed community trajectories (De Cáceres et al. 2019) by calculating the segment lengths between consecutive sampling days to estimate the rate of community change. Here, predator‐containing and predator‐free microcosms were analysed separately due to different sampling intervals. Significant species contributions to community trajectories were identified by projecting their relative abundance as environmental vectors in the PCA via linear regression. A non‐metric permutational ANOVA (PERMANOVA) based on Aitchison distance determined the variables influencing community composition.

### 2.4 Contribution of Species Sorting and Evolution to Trait Change

The relative contribution of species sorting and evolution to prey community trait change was estimated by comparing the observed trait distributions (carrying capacity and defence) to predictions based on species sorting. For each microcosm/sampling time, community trait distributions expected from species sorting were estimated from 24 random draws from the initial species trait pool weighted by species frequency. The evolutionary and ecological contributions to community trait change for each microcosm/sampling time were estimated by calculating the logarithmic ratio of the median observed trait (*n* = 24 clones, see ‘Phenotypic characterisation’) and the median expected trait (*n* = 100 repeats of 24 random draws). Temporal trends were assessed by fitting regressions of the logarithmic ratios against experiment days, deriving intercepts and slopes per microcosm. Molecular evolutionary dynamics were inferred from whole‐genome sequencing of the initial bacterial populations and metagenome sequencing of the (mis‐)match experiment, as described earlier (Hogle et al. 2023). Complete details of the sequencing methods are provided in the Supplementary Information.

## 3 Results

### 3.1 Phenotypic Change

**FIGURE 2:**
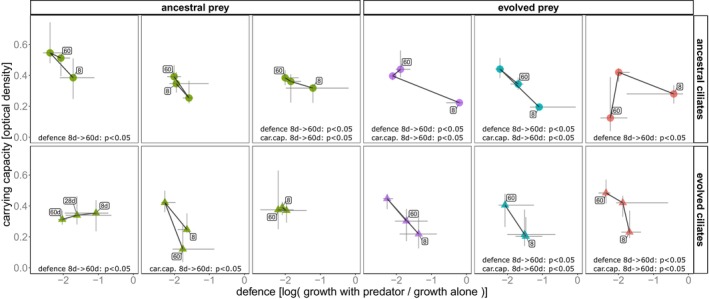
Phenotypic change. Each of the 12 microcosms was sampled on Days 8, 28 and 60 to follow community‐level changes in carrying capacity and anti‐predator defence. On each sampling Day, 24 clones from each microcosm were randomly isolated, and their growth in the absence and presence of the predator was assayed. Carrying capacity was taken as the optical density (600 nm) in the absence of predators measured after 96 h of growth. Anti‐predator defence was calculated as the logarithmic ratio between the OD in the presence and absence of predators. The data points represent community‐level phenotypes calculated as the median traits of the 24 clones per sampling day. Error bars represent 50% quantiles of the trait distributions. Significant differences between Day 8 and Day 60 defence and/or carrying capacity distributions (Kruskall‐Wallis *p* < 0.05) are shown. Data points from Days 8 and 60 are labelled, and a line connects points to indicate the temporal sequence. Data point shape indicates evolutionary history of the predator: Circles = ancestral predators, triangles = evolved predators. Colours represent evolutionary history of the prey: Green = ANC prey, violet/cyan/red = EVO prey from replicates A/B/C.

We assessed community‐level trait changes for carrying capacity and anti‐predator defence (hereafter defence) on Days 8, 28 and 60 of the experiment (SI). Defence levels decreased while carrying capacity increased across all (mis‐)match combinations (Figure 2). Between the beginning and end of the experiment, community defence decreased in eight microcosms, while community carrying capacity increased in five microcosms (Tables S1 and S2; Kruskal‐Wallis test, BH‐corrected *p* < 0.05). Overall, the changes in community carrying capacity were negatively correlated with changes in community defence (median traits on sampling days, Kendall's coefficient = −0.537, *p* < 0.001). In most microcosms, the community's mean phenotypic response changed the magnitude and/or direction on Day 28. Generally, most isolated clones had low levels of defence (Figure S1).

### 3.2 Ancestral and Evolved Prey Communities Differ in Composition

**FIGURE 3:**
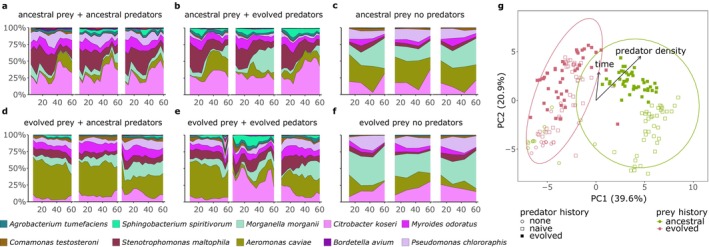
Community composition. (a–f) Bacterial community composition derived from 16 s rRNA amplicon sequencing is shown through time for three microcosms per (mis‐)match combination. Only species with average relative abundance ≥ 1% are denoted. (a, b) Microcosms with ancestral prey communities, (d, e) with evolved prey communities all combined with either (a, d) ancestral (b, e) or evolved predators. (e, f) Microcosms without predation. Microcosms with predators were sampled every 4 days, and control microcosms on Days 12, 28, 44 and 60. (g) PCA (Aitchison distance) of prey community compositions based on the species frequencies from the 16 s rRNA sequencing. Colours indicate the evolutionary history of the bacteria. Predatory density (cells/mL) and increasing time are fitted as environmental vectors and plotted with the ordination (*p* < 0.05).

We compared species compositional differences between replicate communities, (mis‐)match combinations and over time using 16 s rRNA amplicon sequencing. Eight species dominated community composition across all microcosms, while 10 rarer species fluctuated through time depending on the (mis‐)match combination (Figure 3a–f, Table S13). We used PCA (methods) to reveal intrinsic patterns in high‐dimensional community composition (Figure 3g). The evolutionary history of the prey communities separated along PC1 (36.6% variation explained, PERMANOVA *p* = 0.006, Table S3), while predator evolutionary history partitioned along PC2 (20.9% variation explained, PERMANOVA *p* = 0.001, Table S3). However, partitioning by predator evolution was only apparent for ancestral prey communities.

**FIGURE 4:**
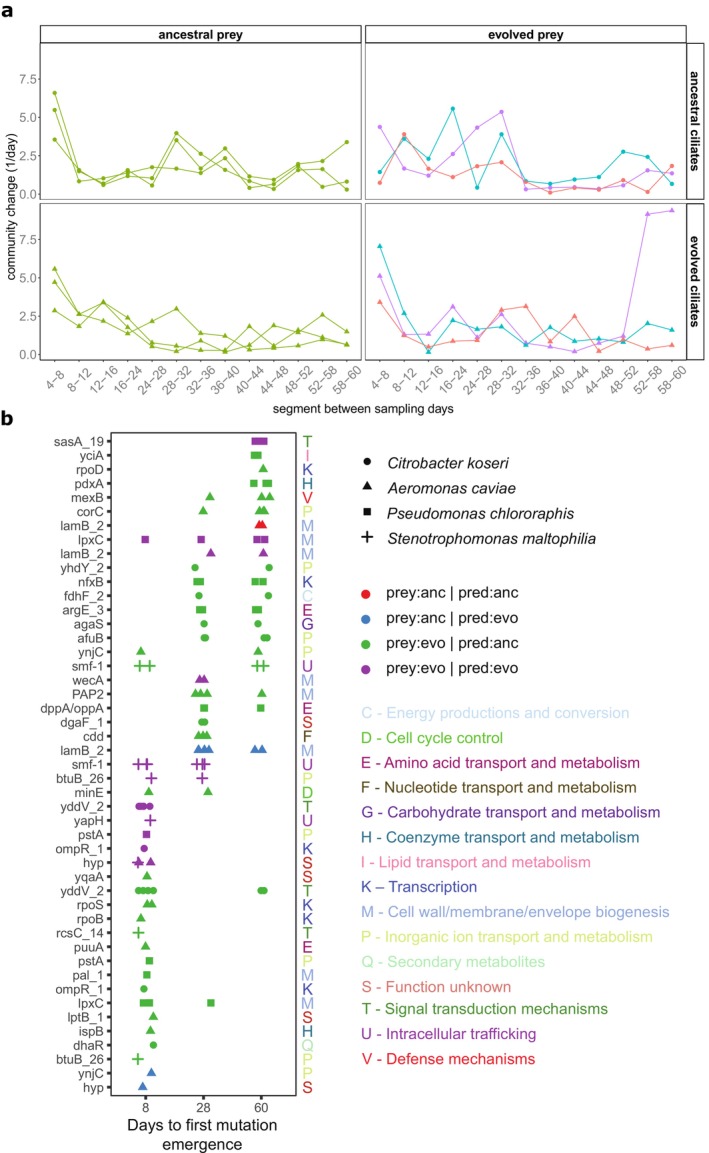
Species sorting and genomic evolution impact community dynamics. (a) Based on the geometric analysis of community trajectories, trajectories were derived by Principal Component Analysis (PCA) on Aitchison distance, and segment lengths between consecutive sampling days in the first two PCA dimensions were calculated and plotted chronologically. As sampling intervals were of equal duration (except Days 16–24), segment length represents the relative speed of compositional change (short: Community change (1/day)). Data point shape indicates evolutionary history of the predator: Circles = ancestral predators, triangles = evolved predators. Colours represent evolutionary history of the prey: Green = ancestral prey, violet/cyan/red = evolved prey lineages A/B/C. (b) Time to *de novo* non‐synonymous mutational emergence. The vertical axis shows the gene name (colour and alphabetically coded by COG functional category at right), while the horizontal axis shows the days when a mutation in that gene was first detected. Point shapes depict species, while colour represents the predator/prey mismatch condition, including all experimental replicates. Only de novo non‐synonymous mutations are included; some genes contain multiple unique mutations.

Using separate PCAs (methods), we further geometrically analysed trajectories of community composition following De Cáceres et al. 2019, focusing on microcosms with predators (with predators: Figure 4a, Figure S2; without predators: Figures S3 and S4). Prey evolutionary histories also separate community trajectories on PC1 with a significant effect of prey history, time and their interaction on community composition (Figure S2, Table S4, PERMANOVA on Aitchison distance; prey history: *p* = 0.001, time: *p* = 0.001, prey history × time: *p* = 0.002). We then estimated the relative speed of compositional change to identify periods differing in their magnitude of compositional changes (Figure 4a). If sampling intervals are equally long, the length of segments between consecutive sampling days is an estimate for the relative speed of compositional change (incomplete data from day 20 was removed from analysis, resulting in a single‐segment Day 16–28 that spans two sampling intervals). Within all communities, we see an initial period of strong species sorting during the first 8–12 days, followed by a period without or little sorting. In most communities, compositional change then increased again and a second period of sorting set in toward the middle of the experiment. After this, rates of change dropped to similar or lower levels than observed after the initial sorting period. Community trajectories are generally directional along PC2, suggesting that community composition responded similarly to environmental conditions (Figure S2, Table S5). We further found 13 species that contributed significantly to trajectories in the PCA (Figure S5, vegan::envfit, *p* < 0.001).

### 3.3 Genomic Evolution

To identify temporal patterns and signs of selection in genomic evolution, we sequenced evolved bacterial populations (A, B, C per species in Figure 1) before (genomes) and during the experiment (metagenomes). We first looked at general mutational dynamics and then focused on parallel mutations occurring across replicates because they are often under strong selection and provide insight into adaptive evolution (Cooper 2018; Wichman et al. 1999). The total number of non‐synonymous mutations (Figure S6a) and accumulated non‐synonymous mutations (*M(t)*, Figure S6b) were saturated by the experiment midpoint, particularly for the *de novo* mutational trajectories. This pattern is consistent with the idea that molecular evolution had neared an equilibrium between mutations accumulating from positive selection on beneficial *de novo* and standing mutations and mutational loss from negative selection and drift. Indeed, much of the standing non‐synonymous variation was purged from the evolved bacteria in the first 8 days (Figure S7). We further assessed the tempo of evolution by quantifying the time for *de novo*, minor alleles to reach the majority allele status (*f*_*max*_ > 0.5). Minor *de novo* alleles generally monotonically increased to reach majority status by Day 28 or 60, implying steady selection for the duration of our experiment (Figure S8).

We then focused our analysis on gene‐level targets of parallel evolution, aggregating mutations within genes and (mis‐)match combinations with more non‐synonymous mutations than expected by chance. We found that selection by the ciliate predator produced significantly parallel mutational profiles (*p* < 1e^−5^). Across replicate populations, species consistently acquired protein‐altering mutations within the same genes, in evolved prey populations both before (Figure S9) and during the community experiment (Figure S10, Table S6). There was no shared functional category common to all parallel genes across species; instead, functional enrichments in parallel genes were species‐specific. Next, we investigated the extent to which parallel mutated genes were shared across the experimental (mis‐)match combinations for each species. All species starting from evolved populations shared mutated genes, but this predominantly reflected shared standing variation. In 4/5 species, no genes were shared between ancestral or between ancestral and evolved prey histories. However, *de novo* parallel mutated genes in *Aeromonas caviae* were shared between (mis‐)match combinations more often than expected by chance (*p* < 0.05), suggesting similar selective pressures on this species across the mismatch combinations.

Finally, we focused on the temporal dynamics of parallel mutated genes (Figure 4b). We observed a functional succession in mutations over time. Many early emerging parallel non‐synonymous mutations occurred in genes with predicted functions in transcriptional regulation (e.g., *dhaR* and *ompR*), particularly of transporters, and modifications to the outer membrane (e.g., *lptB*, *btuB*, *lpxC*, *ompR*, *yfiB*, *yapH*, *smf‐1 and yddV*). In contrast, later emerging mutations were in genes involved in central metabolism and growth regulation (e.g., *yciA*, *dgaF*, *pdxA*, *fdfH*, *rpoD*, *argE*, *agaS* and *cdd*, Table S6).

### 3.4 Estimating Contributions of Species Sorting and Evolution

**FIGURE 5:**
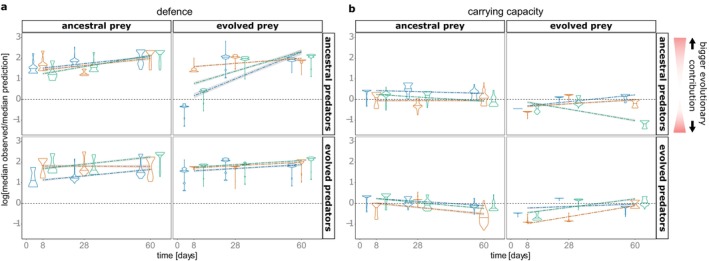
Species sorting and evolution determine phenotypic change. Relative contribution of species sorting and evolution to phenotypic change in the prey communities was estimated by comparing observed community trait distributions (carrying capacity and defence) to predictions based solely on species sorting. Observed distributions were taken from the 24 phenotyped clones isolated per microcosm on Days 8, 28 and 60. Predicted trait distributions are based on the initial trait measures of the bacterial species used to inoculate the experiment. For each sampling day and microcosm, these were computationally estimated by using the respective species frequency from 16 s rRNA amplicon sequencing as the probability to draw the corresponding trait pair (sample size: 24, repeated 100 times). Comparison of observed predicted trait distributions was calculated as the logarithmic ratio between the median observed and the median predicted trait. The greater the relative contribution of evolutionary change for community‐level traits, the further away from zero the values are, and the closer to zero the greater the contribution of species sorting is. Violin plots illustrate the distribution of the 100 resampled ratios including their mean value. To identify temporal dynamics, regressions along Days 8, 28 and 60 were determined for each microcosm (dashed lines, grey area = 95% confidence interval). (a) community anti‐predator defence and (b) community carrying capacity. Colours: Three replicate microcosms per combination of prey and predator evolutionary history: Blue = microcosm 1, yellow = microcosm 2, green = microcosm 3.

To distinguish the relative contribution of species sorting (Figure 4a) and evolution (Figure 4b) to community trait change (Figure 2), we predicted community traits based on species sorting, using the initial species traits (Figure S11) and species frequencies recorded on the sampling days (Supporting Information). We compared these predictions with the observed community traits (Figure S12) and computed the log ratio between the observed and predicted median traits (Figure 5). This log ratio quantifies the contribution of evolutionary phenotypic change relative to phenotypic change by species sorting within the communities. Values further away from zero indicate greater evolutionary change relative to ecological change. When contrasting the two traits, we found a greater relative contribution of species sorting for community carrying capacity, as the predictions are generally close to the observed distribution. Temporal deviations were small with slopes of linear regressions over time either only slightly positive or negative (slopes: Mean = −1.81 × 10^−4^, SD = 1.03 × 10^−2^, in 11/12 microcosms significant at *p* < 0.001, Tables S7 and S8). In comparison, the contributions of evolutionary change were larger for community defence (Figure 5a,b) with predictions deviating strongly from those observed, already at Day 8 in 10 out of 12 microcosms, and increasing over time, implying an increasing contribution of evolution (slopes: Mean = 1.29 × 10^−2^, SD = 1.16 × 10^−2^; in 11/12 microcosms significant at *p* < 0.001).

### 3.5 Population Densities

**FIGURE 6:**
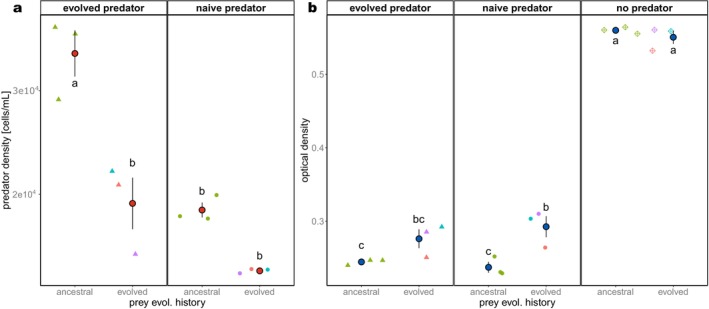
Population densities. Mean population densities of (a) predator and (b) prey in the 12 experimental microcosms and 6 microcosms without predators (3 replicates per evolutionary history of prey). Microcosms were sampled every 4 days. Small symbols: Mean value of all sampling days per microcosm; large symbols (red and blue) represent the mean of the three replicates per combination of predator and prey evolutionary history, and error bars represent the corresponding standard error. Letters illustrate significance (*p* < 0.05) of pairwise comparisons (Tukey's HSD) between combinations of evolutionary history derived from two separate ANOVAs on predator and prey densities (Tables S9 and S10). Groups with the same letter at the top of the box are not significantly different. Data point shape indicates evolutionary history of the predator: Circles = ancestral predators, triangles = evolved predators. Colours represent the evolutionary history of the prey: Green = ancestral prey, violet/cyan/red = evolved prey lineages A/B/C.

To test whether and how predation, evolution and predator or prey evolutionary history impacted population densities, we analysed predator and prey densities averaged across sampling days (Figure 6; two analyses of variance (ANOVA), Tables S9a–c and S10a–c). We found that evolved predators and evolved prey communities had higher population densities than the ancestral communities (predator history on predator density: *F*_(1,10)_ = 39.98, *p* < 0.001; prey history on prey density: *F*_(1,14)_ = 11.5, *p* = 0.005). Additionally, evolutionary history of predator significantly impacted prey density (predator history on prey density: *F*_(2,15)_ = 657.4 *p* < 0.001; predator history X prey history on prey density: *F*_(2,12)_ = 6.2, *p* = 0.014) and prey community evolutionary history significantly impacted predator density (prey history on predator density: *F*_(1,9)_ = 35.4, *p* < 0.001; prey history X predator history on predator density: *F*_(1,8)_ = 6.3, *p* = 0.0361). The mismatch in evolutionary history between predator and prey affected predator but not prey densities (Figure 6a,b). Ancestral predators grown on evolved prey reached the lowest densities, whereas evolved predators grown on ancestral prey had the highest densities, but densities in evolutionary matches were at intermediate levels (Figure 6a). Whether the same prey evolutionary history was matched or mismatched with the predator history did not significantly affect prey density (Figure 6b). Overall, prey communities had higher densities in the absence than in the presence of the predator, but ancestral and evolved prey densities did not differ without the predator.

## 4 Discussion

Tracking community carrying capacity and defence over time, we found that carrying capacity increased and defence decreased in all communities where the predator was present. We also tested the effects of predator and prey evolutionary history on community traits and composition and found that prey history led to different outcomes in community composition and resulted in different patterns of genomic evolution but not trait evolution. The changes in the community‐level traits were driven by two distinct processes, and the relative role of these two processes changed over time; changes in carrying capacity were mainly driven by species sorting, while defence changed mainly through evolution. Our results underline that evolutionary history can impact eco‐evolutionary dynamics in communities (Padfield et al. 2020; Pantel, Duvivier, and Meester 2015; Reznick and Travis 2019; Urban 2013).

### 4.1 Dynamics of Species Sorting and Evolution Drive Community Trait Change

Carrying capacity and predator defence were affected differently by species sorting and evolution. Species sorting was initially effective when selection favoured a higher carrying capacity, and the community was able to respond because trait variation for higher carrying capacity was present in the initial species composition (Figure 2, Figure S11). After community composition stabilised following a first period of species sorting (after Day 8), we observed a significant evolutionary influence, with most phenotypic evolution occurring by Day 28 and genomic evolution building up until Day 28 when the total number of *de novo* non‐synonymous mutations saturated (Figure 2, Figure S6). During this period, selection acted mainly on defence favouring lower levels of community defence. As the initial defence variation in the community did not provide such low levels of defence (Figure 2, Figure S11), the community could only respond by evolving. Following the evolutionary change between Days 8 and 28, we found that species sorting became more important again, favouring a high carrying capacity.

Nonetheless, selection on the evolved variants remained constant as *de novo* alleles kept increasing to reach majority status by Day 60. The temporal pattern of evolution and species sorting was similar for all combinations of prey and predator evolutionary history (Figure 4a). Our results are consistent with recent studies showing that the relative roles of evolution and species sorting can depend on environmental conditions (Bell et al. 2019; Jewell and Bell 2023; Low‐Décarie et al. 2015; Pillai, Gouhier, and Vollmer 2017). Our results also suggest why the two processes affect the two traits differently and why their relative roles changed over time. When variation in the direction of selection is present across species, sorting is the main driver of change, whereas evolution dominates in the absence of variation across species in the direction of selection. This is in line with the general theory on phenotypic change through evolutionary and ecological changes (Fronhofer et al. 2023; Lande 1976; Orr and Unckless 2008) and a recent advance in the integration of ecology and evolution (Vellend 2010). However, direct tests are lacking, and our study cannot provide direct experimental evidence for this.

### 4.2 Evolutionary History and Predation Shape Community Composition

Community assembly of microbial communities is generally complex as species interact directly and indirectly via their environment (Chang et al. 2023). Indeed, predation impacted species sorting, which led to distinct community compositions compared to predator‐free communities (PCA, Figure 3g), with two species ( *Agrobacterium tumefaciens* and *Sphingobacterium spiritivorum* ) being only abundant in the presence of the predator suggesting a competitive advantage mediated by the predator (Table S14). Community composition diverged comparing ancestral and evolved prey communities when under predation (Figure 3g). Predator evolutionary history had only a minor effect on the prey community composition. Even though community composition diverged depending on the evolutionary history, species sorting was almost deterministic. This is illustrated by the directionality of community trajectories across replicates and the high repeatability of the temporal dynamics across replicates and evolutionary histories (Figure S2, Figure 4a). This suggests that all communities responded similar to their environment, even if they started with different trait distributions (Figure S11). Functional and/or taxonomic determinism is often observed in community assembly (Goldford et al. 2018; Louca et al. 2018). Repeatability at the species level is rarer and usually only observed when stochastic processes are negligible (Cairns et al. 2020; Fernandez‐Gonzalez, Huber, and Vallino 2016; Kaewpipat and Grady 2002; Vanwonterghem et al. 2014). Repeatability could also derive from an evolutionary priority effect where some species were pre‐adapted to the conditions in our experiment (De Meester et al. 2016; Nadeau et al. 2021). This could lead to differences in composition between ancestral and evolved communities and also explain why evolved communities differ less between both predator histories (Figure 2g; i.e., pre‐adaptation against predators is effective, irrespective of predator history (Hiltunen and Becks 2014, 20014; Huang et al. 2017)).

### 4.3 Genomic Evolution in Prey Is Determined by Taxa and Its Evolutionary History

Regardless of prey evolutionary history, non‐synonymous mutations tended to cluster in the same genes across replicate populations of each species from the same match–mismatch combinations, implicating strong directional selection from each unique experimental combination. However, we found few targets shared across the match–mismatch combinations, except the shared mutated genes derived from pre‐existing variation. Furthermore, these genes were not organised into shared coarse‐grained functional categories across taxa. Except for A. caviae (see Supporting Information), our results are consistent with prior evolve and resequencing experiments that identified idiosyncratic targets of selection and unique mutational spectra across species evolving in response to the same selective pressure (Shoemaker et al. 2021) as well as evolution under distinct nutritional stresses (Maharjan and Ferenci 2017). Our findings suggest that although molecular evolution in response to predator match–mismatch was highly repeatable for each prey species, this predictability was largely contingent upon the evolutionary history of the prey/predator and was not generalisable at the functional level across prey taxa.

Mutational dynamics in the evolved prey primarily differed from ancestral prey due to standing genetic variation. Some standing non‐synonymous variants rose from intermediate frequencies to approach fixation at the experiment end. This variation may have been adaptive in both the evolved prey populations pre‐experiment and community contexts or hitchhiked on other adaptive clonal backgrounds as part of a mutational cohort (Lang et al. 2013). Other standing non‐synonymous variations were purged during the experiment. These dynamics could reflect chance clonal interference (Gerrish and Lenski 1998) between standing adaptive variants and *de novo* beneficial mutations or potentially reflect deterministic pleiotropic costs from interspecific competition. Thus, standing variation appears to have also played an important role in shaping the overall pace of prey evolution, which likely contributed to altered ecological interactions with the predator and potentially altered subsequent selection (Faillace, Grunberg, and Morin 2022; Lawrence et al. 2012; Raynaud et al. 2022).

### 4.4 Defence in Prey Communities Declines Under Predation

In contrast to the results with single prey under predation, where predation generally leads to an increase in the defence level (Abrams 2000; Hiltunen and Becks 2014; Jousset 2012; Meyer and Kassen 2007; Yoshida et al. 2003), selection by predators reduced the level of anti‐predator defence in the bacterial communities. This suggests that interactions between bacteria were a stronger selection factor and that the increasing carrying capacity may have been driven by selection through competition. In addition, selection for defence may have been masked if the defences conferred by few species protected the entire community (Aijaz and Koudelka 2019; Bertness and Callaway 1994; Jousset 2012; Mazzola et al. 2009). We observed a relatively small subset of clones that was well defended, making this a possible mechanism (Figure S1). The ciliate *Tetrahymena thermophila* has been shown to exhibit preferential consumption when feeding on a similar community which was stronger when the ciliate population was more diverse (Hogle et al. 2022). Such a mechanism can further reduce the selection of defence on the whole prey community. Finally, a communal defence such as growth in biofilms can protect members from predation. As we measured community defence, we would have been able to detect this mechanism in our experiments. Generally, selection by predation and the bacterial community is non‐additive, and despite a significant impact of predation on community dynamics, bacterial interactions divert prey evolution to lower defence (terHorst et al. 2018), thereby reducing predator–prey coevolution resulting in phenotypic mismatches between predators and most prey.

### 4.5 Declining Defence Might Be Linked to Trade‐Off With Growth Rate

Functional inference from genomic data can reveal additional insights into the underlying changes in defence. Enriched functional categories related to inorganic ion transport and cell membrane biogenesis were found in parallel mutations across evolved prey populations pre‐experiment (Figures S9). Most parallel mutated genes were Gram‐negative outer membrane (OM) transporters/porins, their transcription factors or related to modification of the OM surface (e.g., lipopolysaccharides and exopolysaccharides). These proteins are often targets for bacterial enemies such as microbial predators (Mun et al. 2022; Sun, Kjelleberg, and McDougald 2013; Wildschutte et al. 2004). For example, *ompR* regulates the expression of the OM porins OmpC/OmpF (Mizuno and Mizushima 1987) and was mutated in all three *Citrobatcer* populations. In *E. coli* , the ratio of these two porins determines predation susceptibility to *Bdellovibrio* (Mun et al. 2022). Lipopolysaccharides, lipoproteins and O‐antigen‐related genes (*yddV*, *lpxC*, *yfiB*, *rcsC*) also had highly parallel mutations, and these OM structures are known to influence prey recognition by predators (Arnold, Spacht, and Koudelka 2016; Wildschutte et al. 2004). Thus, mutations to genes encoding OM structures acquired during *Tetrahymena* co‐culture likely reflect selection for increased predator evasion via altering the structure and density of molecules on the prey OM.

During the (mis‐)match experiment, parallel mutated genes showed enrichment in functions related to inorganic ion transport and cell membrane biogenesis (Figure S10), highlighting their relevance for prey adaptation in a community context. However, many non‐synonymous parallel mutations in the starting prey populations decreased in frequency (Figures S7), coinciding with the phenotypic loss of defence during the experiment. For example, *ompR* was nearly fixed in parallel evolved *Citrobacter koseri* populations before the experiment but rapidly lost from all populations during the experiment, which may reflect competing selective pressures from defence and nutrient acquisition in a competitive community (Liu and Ferenci 1998, supporting discussion). *De novo* non‐synonymous parallel mutations also emerged during the experiment in some of the same genes mutated in pre‐adapted populations (e.g., *yddV*). Thus, it is likely that different mutations in the same OM‐modifying genes reflect selection for various degrees of prey defence/growth performance trade‐off in single prey cultures versus competitive communities (Ferenci 2016). We anticipated the evolutionary loss of defence to benefit the prey (Ferenci 2005) but found little evidence of a trade‐off between defence and prey carrying capacity. Instead, prey species generally evolved lower predator defence without a significant change in carrying capacity.

The reduction of prey defence may be associated with increased growth rate and not carrying capacity, consistent with theoretical predictions and our experimental observations. In continuous single‐nutrient supplied systems, the species with the lowest nutrient uptake affinity (i.e., highest carrying capacity at the lowest R*) is expected to outcompete others (Tilman 1982). However, batch culture systems are characterised by infrequent nutrient pulses (here every 96 h) where nutrient and cell concentrations vary over orders of magnitude. Here, growth rate increasingly dominates competition, allowing fast‐responders to capitalise on the high nutrient concentrations during a transfer event (Letten and Ludington 2023). When a slower‐growing predator is included in this system, the nutrient pulse will also dilute the predator and temporarily decouple the growth of predator and prey, which should further benefit faster‐growing prey species. Following this prediction, many early emerging *de novo* parallel mutations occurred in genes with predicted functions in transcriptional regulation of transporters and direct modifications to OM transporters (Figure 4), potentially increasing nutrient flux into the cell at the cost of lower predator resistance (Ferenci 2005). Later, parallel mutations emerged in genes related to metabolism and growth (Figure 4, supporting discussion). Taken together, we interpret these broad patterns as reflecting a rapid evolutionary retooling of the bacterial outer membrane and transporter repertoire under increased selection for resource acquisition, followed by changes to metabolism that potentially served to optimise metabolism and growth under batch transfer.

### 4.6 Phenotypic Mismatches Impact Population Densities

The (mis‐)match combination of prey and predator also had effects on average predator and prey community densities and selection imposed by the predator. Predators controlled prey communities independently of the evolutionary history of prey and predator. Predator growth varied with evolutionary history and was particularly sensitive to mismatches with prey history. Overall, previous evolution in predator and prey led to adaptations beneficial in their interaction and carried over to the community context.

### 4.7 Conclusions

This study adds to a growing body of work demonstrating the importance of considering the convergence of ecological and evolutionary timescales for community dynamics (Lion 2018). Our study suggests that the temporal dynamics of the relative roles of ecology and evolution may be driven by the presence of phenotypic/trait variation in the direction of selection and that evolution becomes more important in the absence of variation within the species pool. Consequently, identifying the conditions under which we can predict the convergence of ecological and evolutionary timescales requires an understanding of the direction of selection and the variation in traits (Hermann et al. 2024; Hermann and Becks 2022; Scheuerl et al. 2019) and/or the underlying genetics under selection (Barbour, Kliebenstein, and Bascompte 2022; Blanchet, Fargeot, and Raffard 2023; Pantel and Becks 2023; Yamamichi 2022).

## Author Contributions

T.H. conceived, designed and performed the experiments, J.H., S.H. and L.B. wrote the paper and analysed the data. All authors contributed with materials and/or analysis tools.

## Conflicts of Interest

The authors declare no conflicts of interest.

### Peer Review

The peer review history for this article is available at https://www.webofscience.com/api/gateway/wos/peer‐review/10.1111/ele.70033.

## Supporting information


Data S1.

Table S6.

## Data Availability

Data and code for the analyses of this study are openly available on Zenodo, and raw sequencing data is available at NCBI Bioproject number PRJNA1179357. Julius Hoffmann: https://doi.org/10.5281/zenodo.14202345. Shane Hogle: https://doi.org/10.5281/zenodo.14017295. The manuscript is available as a preprint on Research Square: https://doi.org/10.21203/rs.3.rs‐4647074/v1.
