# Supplementary material for: Temporal Changes in the Role of Species Sorting and Evolution Determine Community Dynamics
Source: Ecol Lett. 2024 Dec 31;28(1):e70033. doi: 10.1111/ele.70033 (PMC11687414; doi:10.1111/ele.70033)
Supplement: Supplementary file 1 — Data S1. [file ELE-28-0-s001.pdf]

Supporting Information for

Temporal changes in the role of species sorting and evolution determine community dynamics

Julius Hofmann, Shane Hogle, Teppo Hiltunen, Lutz Becks

This file includes (in this order):

Material & Methods

Bacterial Community
Table S11 Bacterial species in community
Predator Origin
Table S12 Bacterial species in predator selection
Experimental design
Media composition and culture conditions
Phenotypic characterisation
16s-rRNA amplicon sequencing
Community composition
Genome sequencing of prey populations and communities
Genomic data analyses
Contribution of species sorting and evolution to trait change
Population densities
Software

Supporting Discussion
Genomic evolution

Supporting Results
Community composition (Tables S13, S14, S15)
Traits in evolved prey populations

Supporting Figures

Figure S1 Distribution of defence in matches and mismatches
Figure S2 Community trajectories with predators
Figure S3 Predator free community trajectories
Figure S4 Predator free community change
Figure S5 Species contribution to composition
Figure S6 De novo mutational trajectories saturate with time
Figure S7 Heatmap presentation of parallel mutational trajectories
Figure S8 Time to major allele status
Figure S9 Functional enrichments in parallel mutated genes from starting evolved populations

Figure S10 Functional enrichments in parallel mutated genes during the community experiment
Figure S11 Initial traits
Figure S12 Contribution of species sorting and evolution to phenotypic change
Figure S13 Community inocula
Figure S14 Higher allele frequencies in parallel mutated gene

Supporting Tables

Table S1
Table S2
Table S3a-c
Table S4
Table S5
Table S7
Table S8
Table S9a-c
Table S10a-c
Table S15

Other supplementary material for this manuscript:

Table S6 List of parallel mutated genes

Material & Methods

Bacterial Community

Ancestral prey communities were assembled from clonal species derived from a culture collection (Microbial Domain Biological Resource Centre (HAMBI mBRC), University of Helsinki, Finland). See Table S11 for a list of all species. Prior to the experiment, all bacterial stocks were kept at –80°C.

Evolved prey (for evolved communities) were derived from co-cultures with ancestral predators. For these, the ancestral prey species were each separately cultured in triplicates (A,B,C in Fig. 1a) with ancestral predators for 100 days. The co-cultures were maintained in semi-continuous cultures with 3% transfer to fresh medium every 4 days (approx. 165 generations, further detail below). Resulting evolved populations were then freeze-stored in Glycerol (see media composition) until community assembly.

Evolved prey selection:

Co-cultures of ancestral prey species with ancestral predators to yield the evolved prey populations were performed at 25°C in 6ml 5% King's B (KB) medium (see media composition) under shaking (70rpm), with 3% transfer to fresh medium every 4 days .

Table S11. HAMBI species in the prey community. The table lists all 24 species within the bacterial prey community. Strain IDs from the HAMBI collection are listed alongside full species names.

strainID	genus	species
HAMBI-0006	*Pseudomonas*	*putida*
HAMBI-0097	*Acinetobacter*	*lwoffii*
HAMBI-0105	*Agrobacterium*	*tumefaciens*
HAMBI-0262	*Brevundimonas*	*bullata*
HAMBI-0403	*Comamonas*	*testosteroni*
HAMBI-1279	*Hafnia*	*alvei*
HAMBI-1287	*Citrobacter*	*koseri*
HAMBI-1292	*Morganella*	*morganii*
HAMBI-1299	*Kluyvera*	*intermedia*
HAMBI-1842	*Sphingobium*	*yanoikuyae*
HAMBI-1896	*Sphingobacterium*	*spiritivorum*
HAMBI-1923	*Myroides*	*odoratus*
HAMBI-1972	*Aeromonas*	*caviae*
HAMBI-1977	*Pseudomonas*	*chlororaphis*
HAMBI-1988	*Chitinophaga*	*sancti*
HAMBI-2159	*Paraburkholderia*	*caryophylli*
HAMBI-2160	*Bordetella*	*avium*
HAMBI-2164	*Cupriavidus*	*necator*
HAMBI-2443	*Paracoccus*	*denitrificans*
HAMBI-2494	*Paraburkholderia*	*kururiensis*
HAMBI-2659	*Stenotrophomonas*	*maltophilia*
HAMBI-2792	*Moraxella*	*canis*
HAMBI-3031	*Niabella*	*yanshanensis*
HAMBI-3237	*Microvirga*	*lotononidis*

Predator origin

Ancestral predators were clonal isolates of *Tetrahymena thermophila* strain 1630/1U (CCAP) mating-type (type II) that had been serially propagated in axenic culture (without bacteria) under conditions restricted to asexual reproduction in proteose peptone yeast extract (PPY) medium (see media composition) (Collins, 2012). Evolved predators were mixed from *Tetrahymena thermophila* populations derived from a previous LTEE. Briefly, ancestral *Tetrahymena thermophila* populations were co-cultured with each one of seven bacterial species (for each species in three replicate lines, NOTE: with the exception of

Comamonas testosteroni these species were not part of the 24 species in the community, see Supp. Table 12). LTEE lines were kept in batch cultures (6 ml of 5% KB medium, 1% weekly transfer) and after 600 predator generations, Tetrahymena populations were isolated from all lines using an antibiotic cocktail (kanamycin, rifampicin, streptomycin and tetracycline). Evolved ciliate populations were then freeze-stored in liquid nitrogen using a modified protocol from (Cassidy-Hanley, 2012) (see (Cairns et al., 2020)). Prior to the community (mis-)match experiment evolved ciliates from all populations were revived from storage, grown to high density in PPY medium and then combined in equal densities to form the evolved predator population in this experiment.

Table S12. Prey species in predator selection. The seven species that were each cultured (in triplicates) with ancestral predators for 600 predator generations to yield evolved predators. All evolved predator populations were combined to form one single evolved population. ATCC, American Type Culture Collection; HAMBI, HAMBI mBRC, Microbial Domain Biological Resource Centre HAMBI, University of Helsinki, Finland.

strainID	genus	species
ATCC 11303	*Escherichia*	*coli*
HAMBI 1919	*Janthinobacterium*	*lividum*
HAMBI 103	*Sphingomonas*	*capsulata*
HAMBI 18	*Brevundimonas*	*diminuta*
SBW25	*Pseudomonas*	*fluorescens*
HAMBI 403	*Comamonas*	*testosteroni*
ATCC 13880	*Serratia*	*marcescens*

Experimental design

For community assembly ancestral and evolved bacterial populations were revived from Glycerol stocks and then grown in fresh 5% KB medium for 48h. All cultures were then adjusted to OD 1. Equal volumes of ancestral species were combined in a single ancestral community inoculum whereas the three evolved community inocula were assembled in equal volumes from each one of the three evolved selection lines per species (A,B,C in Figure 1). Prey communities and the predator populations of both evolutionary histories

were combined in a fully factorial design, including microcosms without predators. Three microcosms (biological replicates) for each combination of prey history with a predator condition (i.e. ancestral predators, evolved predators, no predators) were established in laboratory microcosms. These Community (mis-)match experiment microcosms were established in 25mL glass flasks containing 6 mL 5% KB medium. Cultures were kept at 25°C under shaking (70rpm) with 30% transfer (1800μl) to 4.2mL fresh 5% KB medium every 4 days. At each transfer 1ml of culture was taken for further analysis (phenotypic characterisation 16s rRNA amplicon sequencing, WGS metagenomics sequencing, population densities). Cultures were maintained for 60 days (14 transfers).

Media composition

King's B medium:

5% King's B medium was prepared from 1g Proteose peptone N°3, 0.5ml 85% Glycerol and 11.28 5x M9 salts per 1l deionized water.

Proteose Peptone Yeast extract (PPY) medium:

PPY was prepared with 20 g of proteose peptone and 2.5 g of yeast extract in 1 l of deionized water.

Frozen bacterial stocks:

All bacterial stocks were freeze-stored at -80°C with 0.5 ml of culture mixed with 0.5 ml 85 % Glycerol.

Phenotypic characterisation

Carrying capacity and defence of ancestral and evolved prey species were assayed before community assembly. For this a population of each species and evolutionary history

(ancestral or evolved) was plated on growth agar and 8 clones were randomly picked to be phenotyped. During the experiment the corresponding community level changes in these traits were followed by sampling each predator containing microcosm at day 8, 28 and 60 (Figure 2). On each sampling day 24 clones were isolated from each microcosm (see below) and clonal carrying capacity and defence was assayed (see growth assays). Community carrying capacity and defence were then calculated as the median values of the 24 clones per sample and day.

Clonal isolation during the Community (mis-)match experiment:

On each sampling day 24 clones were isolated from each microcosm by plating the diluted samples on growth agar (15g/L agar + 50% PPY medium) and picking 24 random colonies (after 24h). These clonal populations were then cultured overnight in 200µl PPY at 25°C under constant shaking and freeze stored in glycerol at -80°C (0.5 ml of culture in 0.5 ml 85 % glycerol).

Growth assays:

For growth assays, 100µl of freeze-stored bacterial stocks were revived in 2ml of 5% KB medium and acclimatised for several generations under constant shaking at 25°C for 24h. These overnight cultures were pin-replicated in fresh 5% KB medium on Bioscreen-compatible honeycomb plates and incubated at 25°C under constant shaking. For all populations/clones bacterial growth in absence and presence of ancestral predators was followed by measuring optical density (600nm) over 96 hours using a Bioscreen C well-plate reader (Oy Growth Curves AB Ltd., Turku, Finland). Starved (see below) ancestral *Tetrahymena thermophila* were added to predator containing samples at an in-sample concentration of 1000 cell/ml. Carrying capacity was taken as the OD in absence of predators after 96h ($OD96_{-P}$). Defence was calculated as the logarithmic ratio between the OD in presence ($OD96_{+P}$) and absence of predators (defence = $\log(OD96_{+P}/OD96_{-P})$).

Starving *Tetrahymena thermophila* before growth assays:

Before growth assays to estimate bacterial defence, *Tetrahymena* were cultured in fresh PPY for 5 days, after which the medium was removed by centrifugation (2x 8min at 3300r.p.m. in +4°C) and the ciliates were starved in M9 salt solution overnight. Starved *Tetrahymena* were then added to samples at an in-sample concentration of 1000 cell/ml.

Statistical evaluation:

Carrying capacity and defence distributions on day 8 were statistically compared to those on day 60 via individual Kruskal-Wallis tests (stats::kruskall.test) for each microcosm and trait (24 clones per day and microcosm). Corrected p-values were derived by performing Benjamin-Hochberg procedure for multiple testing (Tables S1,S2).

16s-rRNA amplicon sequencing

16s rRNA amplicon sequencing was performed on ancestral and evolved starting inocula to confirm presence of all bacterial species (Figure S13). Generally, species abundances in starting inocula do not help to explain later community composition as species prevalent in the inocula can end up being rare in the prey community (e.g. HAMBI 2792, 1299, 0097,1842) and rare ones can end up common (e.g. HAMBI 1292, 2659, 1977). Starting from day 4 all microcosms were sampled for 16s rRNA amplicon sequencing (Figure 3a-f). Microcosms with predators were sampled every 4 days and control microcosms without predators on day 12, 28, 44 and 60. The DNeasy 96 Blood & Tissue Kit (Qiagen) was used to extract DNA from 500 µL of cryopreserved samples (40 % glycerol). The Illumina 16S Metagenomic Sequencing Library Preparation protocol (Illumina, San Diego) was followed to amplify the V3–V4 hypervariable region of the 16S rRNA gene from community DNA using the primer pair CR1_Forward (50 bp): 5'–

TCGTCGGCAGCGTCAGATGTGTATAAGAGACAGCCTACGGGNGGCWGCAG–3’, PCR1_Reverse (55 bp): 5’–GTCTCGTGGGCTCGGAGATGTGTATAAGAGACAGGACTACHVGGGTATCTAATCC–3’ in a limited-cycle PCR reaction followed by dual-indexed Nextera XT barcodes. Libraries were sequenced on the Illumina MiSeq using paired 300 bp reads and MiSeq v3 reagents at the Finnish Institute of Molecular Medicine (FIMM). Bcl2fastq2 v2.2 was used to demultiplex samples, and paired-end reads were quality controlled, merged, filtered, and mapped to the known 16S sequences of the 24 prey species, as described in detail earlier (Hogle et al., 2022).

Community composition

Compositional differences between microcosms were first analysed by a Principal Component Analysis (PCA) (pca function in R package ape) based on the Aitchison distance between communities in all microcosms (including those without predators) on all sampling time points (vegdist function in R package vegan (Oksanen, Jari et al., 2022)) (Figure 3g). Predator density (cells/mL) and time (sampling days) were fitted as environmental variables ($p < 0.05$) using the function envfit in the R package vegan. In a second step we followed the framework based on geometric analysis of community trajectories developed by (De Cáceres et al., 2019) (Figures 4a, S2, S5). Community trajectories were derived via PCA (as above), visualised in two PCA dimensions and segment lengths between consecutive sampling days were calculated via the trajectoryLengths function from the R package ecotraj (De Cáceres et al., 2019; Sturbois et al., 2021). As sampling intervals were of equal duration (except day 16 – 24, where data was missing on day 20), segment length represents relative speed of compositional change. Predator-containing microcosms (Figures 2, 4a) were analysed separately from predator free microcosms (Figures S3, S4) because the latter were sampled at fewer

intervals. Species with significant contribution to community trajectories were identified by fitting their relative abundance as environmental vectors to the PCoA ordination (linear regression via envfit function R package vegan) (Figure S5).

Further, a non-metric permutational ANOVA (PERMANOVA) based on Aitchison distance was performed to determine which variables drove community composition (ADONIS2 function R package vegan) (Table S4). Time, predator, and prey evolutionary history (including their interactions) were used as explanatory variables and we corrected for temporal pseudo-replication (permutation within microcosms (experimental units) set to type “series”).

Genome sequencing of prey populations and communities

Whole genomes of pre-experiment *Tetrahymena* co-cultured prey populations were sequenced before the start of the Community (mis-)match experiment. Cultures of predator-evolved prey populations were grown overnight in PPY medium, cells were pelleted, and genomic DNA extracted using the DNeasy 96 Blood & Tissue Kit (Qiagen), sequencing libraries prepared using the tagmentation-based Nextera Flex (Illumina, San Diego) protocol with dual-indexed multiplexing, and sequencing was performed on an Illumina MiSeq (PE 300bp, v3) at FIMM. To follow genomic evolution during the Community (mis-)match experiment, predator containing microcosms were sampled at days 8, 28 and 60 to sequence community DNA (metagenomes). Community DNA was extracted from cryopreserved mixed community samples directly using the DNeasy 96 Blood & Tissue Kit, libraries were prepared using the Nextera Flex protocol with dual-indexed multiplexing, and paired 100-bp reads were generated on an Illumina HiSeq 2500 in rapid mode at FIMM. Sample demultiplexing was done with Bcl2fastq2 v2.2 and quality/adapter trimming was done using fastp v0.23.4 (Chen, 2023) with default parameters.

Metagenome reads were first processed using competitive read mapping against the collection of closed reference genomes using bbsplit (sourceforge.net/projects/bbmap/), which maps reads to multiple reference genomes using bbmap and determines the best-matching genome for each read pair. We discarded all read pairs that mapped ambiguously (i.e., multiple mapping positions within a containment threshold of the top scoring mapping position) to more than one reference genome but retained reads mapping ambiguously within a genome (e.g., from repeat regions). This produced a collection of read pairs specific to each species from each metagenome. At this stage, species with estimated metagenome coverage $< 5x$ were discarded. Next, reads from single-species populations and the species-specific reads binned from metagenomes were mapped to closed

reference genomes for each species (Hogle et al., 2024) using BWA-mem v0.7.17 (Li, 2013). The resulting alignment files were pre-processed for variant discovery (SNVs and short indels) with the GATK v4.4 software suite (van der Auwera & O'Connor, 2020) following GATK best practices workflows. Genomic variants were called using mutect2 (Benjamin et al., 2019) from GATK v4.4 with default parameters for somatic SNVs and indels (e.g., "tumor only mode"), and variants were filtered using FilterMutectCalls in "microbial mode" which optimises filters for polyclonal microbial samples. The resulting mutect2-filtered variants were annotated for functional effects using SnpEff v4.3 (Cingolani et al., 2012) with gene calls from Prokka v1.14.6 (Seemann, 2014). Functional annotations of genes were derived from the eggNOG 5.0 database (Huerta-Cepas et al., 2019) using eggNOG-mapper v2.1.10 (Cantalapiedra et al., 2021). COG category (Galperin et al., 2021) assignments propagated from eggNOG were used to group genes at higher levels of biological organisation for functional enrichment analysis.

Genomic data analysis

Additional hard filters for called genomic variants were implemented at the data analysis stage to reduce the incidence of spurious calls. Specifically, all genomic regions predicted to belong to a mobile genetic element (e.g., prophage, transposable element, mobilizable plasmids) were excluded. Predicted mobile regions were annotated using MGEfinder v1.0.6 (Durrant et al., 2020) and geNomad v1.7.4 (Camargo et al., 2023). SNP clusters containing three SNPs within a sliding 35 base pair window and SNPs within ten bases of an indel to reduce false positives (Bush, 2021) were also excluded. Finally, variants were combined into temporal trajectories by unique species, replicate, and treatment combinations retaining variant trajectories with at least one observation of $f_{alt} > 0.05$ and position depth > 5 and at least one variant passing mutect2 filters. Only nonsynonymous (i.e., amino acid altering) mutations were used for all statistical analyses with gene parallelism and convergent

evolution. The coverage of the alternative allele relative to the total depth of coverage for reads passing the mutect2 internal quality control metrics was used to infer mutational frequencies (f_m) at each particular site in the genome. The cumulative mutational profiles were estimated as the sum of derived allele frequencies for a species/condition at time point t, $M(t) \equiv \sum_m \widehat{f_{mt}}$

Genetic parallelism was calculated following the statistical approach developed and described by Good (Good et al., 2017) and used elsewhere (Shoemaker et al., 2021). Briefly, parallelism can be interpreted as the degree to which nonsynonymous mutations cluster within a gene over what would be expected due to chance and the number of one-fold, two-fold, and three-fold degenerate sites available in a gene (multiplicity). Multiplicity of gene *i* is defined as $m_i = n_i \cdot \frac{L}{L_i}$ where n_i is the number of mutations and L_i is the number of non-four-fold degenerate sites in the gene (i.e., sites with potential for amino acid change) in gene *i* and *L* is the average number of non-four-fold degenerate sites per gene. Under the null, all genes have equal multiplicity $\underline{m} = \frac{n_{tot}}{N_{genes}}$. Genome-wide gene parallelism, $\Delta l = \sum_i n_i \, log \left(m_i/\underline{m}\right)$, is then calculated as the net increase of the log-likelihood of the alternative hypothesis relative to the null across the entire genome. Further details of this approach, including how specific parallel genes were identified, are available from the primary sources (Good et al., 2017; Shoemaker et al., 2021), in the supplementary material, and in the computational notebooks associated with the project git repository.

A simulation-based approach and the Jaccard index were used to determine whether the same significantly parallel genes were identified across multiple match-mismatch conditions more than would be expected by chance. A similar resampling approach to that of Shoemaker (Shoemaker et al., 2021) was used to randomly draw gene sets (I_i, I_j) from every pairwise species by match-mismatch combination, where the sample size was the

number of significantly parallel genes identified in each combination and the probability of sampling a gene was again proportional to its length. The resampling procedure was repeated for 100,000 iterations. The Jaccard index $J(I_i, I_j) = |I_i \cap I_j| / |I_i \cup I_j|$ was calculated for each resampling and compared to the observed $J(I_i, I_j)_{obs}$ resulting in an empirical *P*-value for the number of null simulation results $J(I_i, I_j)_{null}$ above/below the observed. This estimate was used to infer whether the intersection of the set of genes was greater (convergence) or less than (divergence) expected by chance.

For single species/condition combinations, enrichment of individual COG categories in significantly parallel genes relative to the COG profile of the rest of the genome (i.e., genomic background) was inferred using the hypergeometric test. Correction for multiple testings was done by controlling the False Discovery Rate (FDR) via q-values (Storey & Tibshirani, 2003) with a maximum FDR threshold of 5%.

Contribution of species sorting and evolution to trait change

Relative contribution of species sorting and evolution to community trait change in the prey communities was estimated by comparing observed trait distributions (carrying capacity and defence) to predictions based on species sorting (Figures 5, S12). Observed distributions were taken from the 24 clones isolated from the microcosms on day 8, 28 and 60. Predicted trait distributions are based on these traits in ancestral and evolved bacterial starting populations (Figure S11). These trait pairs were computationally sampled (sample size: 24, repeated 100 times) using the relative species frequency on each sampling day in the given microcosm (16s rRNA amplicon sequencing) as the probability to draw them. Samples of size 24 were taken to mimic the sampling of microcosms during the experiment. Evolutionary and ecological contributions to community trait change were then estimated by calculating the logarithmic ratio between the median observed and the median predicted

trait (100 ratios per microcosm, sampling day and trait). Temporal trends of these contributions were then investigated by fitting regressions (per replicate, stats::lm) explaining the logarithmic ratios (dependent variable) by day of experiment (explanatory variable). and we derived intercept and slope for each microcosm (Tables S7,S8).

Population densities

Population densities of predators and the prey community were measured every 4 days during the experiment (Figure 6). To estimate predator densities 100µl of sample were fixed with 50µl of 1:10 diluted Lugol's solution on a 96-well culture plate. Images of fixed samples were then acquired using an Olympus CKX41 microscope and manually enumerated. Prey densities were estimated by measuring sample OD at 600nm using a multi-well plate reader (Tecan Infinite M200, Tecan Group Ltd., Männedorf, Switzerland).

Mean predator density and mean prey OD within microcosms were statistically analysed by two separate generalised linear models (GLM, stats::glm in R, method = "gaussian", link = "identity") to identify how they are affected by combinations of predator and prey history The GLMs were evaluated via an analysis of deviance (equivalent to ANOVA using a normal linear model). Predator and prey evolutionary history and their interaction were used as fixed effects to explain the dependent variables mean predator density or mean OD (Tables S9a-c, S10a-c).

Software

Unless specific software is mentioned, all analyses were performed using R Statistical Software (v4.3.1; R Core Team 2023). For visualisation and data handling further R packages were used: tidyverse (Wickham et al., 2019), multcomp (Hothorn et al., 2008) for compact letter design of pair-wise comparisons of predator and prey densities, patchwork (Pedersen, Thomans Lin, 2022), RColorBrewer (Neuwirth, Erich, 2022), ggh4x (van den Brand, Teun,

2023), ggrepel (Slowikowski, 2023), data.table (Dowle, Matt & Srinivasan, Arun, 2023), gridExtra (Auguie, Baptiste, 2017).

Supporting Discussion

Genomic evolution

Below we provide a detailed description of our findings on parallel genetic changes in evolved prey pre-experiment, and ancestral and evolved prey during the community (mis-)match experiment. Targets of parallel evolution are often under strong selection and provide insight into adaptive evolution (Cooper, 2018; Wichman et al., 1999). Thus, we consider targets of parallelism as candidates for positive selection, which may provide insight into the evolutionary dynamics of our community mismatch experiment. We looked for parallel mutations (occurring across independent replicates) at the nucleotide and gene levels. We sequenced the three coevolved bacterial populations for each species used to start the community mismatch experiment. Additionally, we sequenced community genomes (metagenomes) from three replicates of all treatment combinations in the mismatch experiment on days 8, 28, and 60. In practice, we consider populations of the five highest recruiting species from the metagenomes because only they had sufficient read depth and coverage for genotyping.

After filtering spurious mutations (SI methods), we detected mutations across the three evolved prey populations of all 22 sequenced genomes pre-experiment, 12 of which had identical mutations (same position, same alternative allele) across at least two replicates. Six species had identical mutations in all three populations. To put these observations in context, we compared them to a null model in which mutations were uniformly distributed across the sites in the genome, checking how many repeat hit sites we would expect by chance. Generally, the null model predicts fewer than two parallel

mutations across the three replicate populations, meaning that for 12 species, we observed excess parallelism at the level of individual nucleotide changes. However, multi-hit sites constituted a small fraction of the total observed mutations across all species (~ 3% across species with multi-hit sites).

We focused our analysis on the gene level because we observed relatively few parallel nucleotide changes. We aggregated genes within genomes/treatments with more nonsynonymous mutations than expected from a null model where the mutational distribution is driven solely by gene length (SI methods). This null model was rejected ($P < 1e-5$) for all 22 coevolved species, indicating that nonsynonymous mutations tended to cluster in the same genes across replicate coevolved populations. We also rejected the same null model for the five species we could reliably genotype in the community mismatch experiment. We found that the degree of parallelism increased with increasing maximum allele frequency across experimental treatment combinations and species (Figure S6), which is consistent with the idea that mutations with higher frequencies are primarily driven by strong positive selection across these treatments, whereas low-frequency mutations may be a consequence of hitchhiking (Buskirk et al., 2017), epistasis, and clonal interference (De Visser & Rozen, 2006; Gerrish & Lenski, 1998).

We then searched for candidate parallel mutated genes that deviated from the null model at the individual gene level (SI methods). We identified 62 genes from 15 species in the coevolved populations and 70 genes from four species in the community experiment. We next asked whether functional categories (COGs) were enriched in these sets of genes. No COG category was significantly enriched across all species (permutation test, $P \sim 0.4$). Still, we identified species-specific COGs enriched in parallel mutations in both the pre-adapted populations (Figure S9) and the match/mismatch experiment (Figure S10). In

particular, genes from cell cycle control, cell membrane biogenesis, inorganic ion transport, and intracellular trafficking had more parallel mutations than expected by chance.

Next, we asked to what extent species' genes with parallel mutations were shared across different experimental treatments from the community mismatch experiment. All evolved prey treatments had trivially convergent parallel mutated genes because they started from the same source of standing genetic variation. However, *de novo* parallel mutated genes from one species (*Aeromonas caviae*) were consistently shared between experimental treatments more often than expected by chance ($P < 0.004$). In particular, parallel mutated *A. caviae* genes from ancestral prey treatments significantly overlapped with those in evolved prey treatments, which was driven entirely by mutations to the outer membrane maltoporin LamB, a putative thiamine transporter YnjC, and a hypothetical protein within a flagellar gene operon. Mutations in *lamB* were also present in the standing genomic variation of the coevolved *A. caviae* populations.

In the discussion, we noted that the *ompR* gene was fixed or nearly fixed in almost all evolutionary replicates. The porins OmpC/OmpF (regulated by *ompR*) are important for carbon and nitrogen nutrient uptake and are strongly upregulated in *E. coli* when grown at high growth rates and low glucose concentrations (Liu & Ferenci, 1998). Thus, the loss of parallel mutations in *ompR* may reflect competing selective pressures from defence and nutrient acquisition when C. koseri was grown in a prey community. In discussion we also referred to a distinct mutational increase within genes related to growth and metabolism from the middle to the end of the experiment. In *Aeromonas caviae*, for example, standing variation in *rpoS* (a regulator of the general stress response) was lost while *de novo* mutations emerged in *rpoS* and later in *rpoD* (a metabolic housekeeping sigma factor) (Figure S7). The ratio of RpoS/RpoD has been shown to control the metabolic capabilities of *E. coli*, with lower RpoS/RpoD ratios reflecting increased growth (King et al., 2004) and

metabolic activity (Saxer et al., 2014) mainly due to the dramatic downregulation of transporters by *rpoS* and the bacterial general stress response (King et al., 2004). Other later emerging metabolic genes include *pdxA* (*Pseudomonas chlororaphis*, positive growth rate regulation (Pease et al., 2002)), *argE* (*P. chlororaphis*, acetylornithine deacetylase, branchpoint in the synthesis of polyamines), and *dgaF* (*C. koseri*, d-Glucosaminate uptake and catabolism (Miller et al., 2013)).

Supporting Results

Community composition

In support of our analysis of the community composition (Fig. 3, Fig. 4a) we determined which species were common (>1% rel. abundance on average) and which species were common only in predator presence. We also investigated which went extinct at the end of the experiment. The following tables give the proportion of microcosms (communities) for which the condition above was true. Additionally, community composition (16s rRNA amplicon sequencing) in the starting inocula of the community (mis-)match experiment is displayed.

Table S13. Common species. Listed are those species that reached an average relative abundance of >1% in at least one microcosm. The proportion of all microcosms (18) in which this condition was true is given. 8 species can be considered common in most microcosms. HAMBI-1896 is common in roughly half of the microcosms.

strainID	frac_replicates_common
HAMBI-0105	0.7222222
HAMBI-0403	0.7777778
HAMBI-1287	1.0000000
HAMBI-1292	1.0000000
HAMBI-1896	0.5555556
HAMBI-1923	1.0000000
HAMBI-1972	1.0000000
HAMBI-1977	1.0000000
HAMBI-2160	0.1111111
HAMBI-2659	1.0000000

Table S14. Common species depending on predator presence. Listed are those species that reached an average relative abundance of >1% in at least one microcosm. The data was split between ancestral, evolved and no predator microcosm and the proportion of microcosms in which this condition was true is given. Blank cells mean that the condition was not met in any microcosm. HAMBI 0105 and 1896 are common in predator presence but rare in predator absence.

strainID	Anc.predator	Evo.predator	no_predator
HAMBI-0105	1.0000000	1.0000000	0.1666667
HAMBI-0403	0.8333333	0.6666667	0.8333333
HAMBI-1287	1.0000000	1.0000000	1.0000000
HAMBI-1292	1.0000000	1.0000000	1.0000000
HAMBI-1896	0.6666667	1.0000000	
HAMBI-1923	1.0000000	1.0000000	1.0000000
HAMBI-1972	1.0000000	1.0000000	1.0000000
HAMBI-1977	1.0000000	1.0000000	1.0000000
HAMBI-2160	0.3333333		
HAMBI-2659	1.0000000	1.0000000	1.0000000

Table S15. Species absence at the end of the experiment. Listed are those species that went extinct in at least one microcosm. The proportion of microcosms (total 18) in which a species went extinct is given. Several rare species persisted in the communities until the end. At the end of the experiment, six rare species were detected at low frequencies in at least half of the microcosms (5 in the table and 1 rare species that never went extinct). Four rare species were detected in 1-6 microcosms. Only six species were not detected in any microcosm at the end of the experiment.

strainID	fraction_replicates_absent
HAMBI-0006	0.11111111
HAMBI-0097	0.94444444
HAMBI-0105	0.05555556
HAMBI-0262	1.00000000
HAMBI-1842	1.00000000
HAMBI-1896	0.38888889
HAMBI-1988	1.00000000
HAMBI-2159	0.66666667
HAMBI-2160	0.38888889
HAMBI-2164	0.77777778
HAMBI-2443	1.00000000
HAMBI-2494	0.44444444
HAMBI-2792	0.94444444
HAMBI-3031	1.00000000
HAMBI-3237	1.00000000

Traits in evolved prey populations

The ancestral species responded in two opposite directions to the 100-day selection period with predators (Figure S12). While 10 of 24 species evolved higher defence than their ancestors, 17 of 24 species evolved higher carrying capacity than their ancestors. Only 4 species were able to improve both traits while for the other species improving either trait mostly traded off with decline in the other. Thus, it appears that some species responded to predation by evolving higher defence, whereas others responded to the culture conditions (e.g. the 3% transfer) by evolving higher carrying capacity, as the main selection pressure. However, initial traits and/or the response to the selection period do not explain whether species are abundant during the community (mis-)match experiment.

Supporting Figures

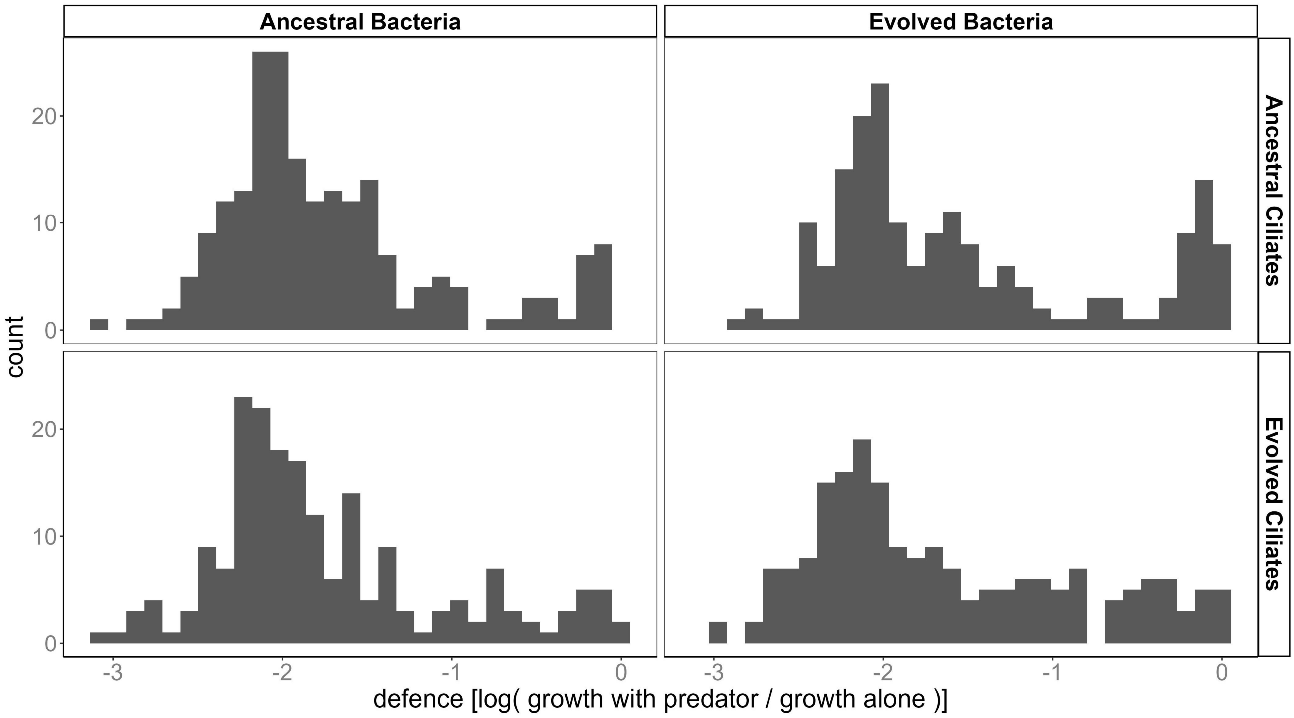

Figure S1 Distribution of clonal defence in matched and miss-matched combinations. For each combination of predator and prey history, clonal defence values were pooled across microcosms and sampling days and visualised in separate histograms (30 bins). Most clones had weak defence but a smaller subset of clones with high defence was present in all combinations. This is consistent with the possibility that only a small part of the community confers a community wide defence (associative defence).

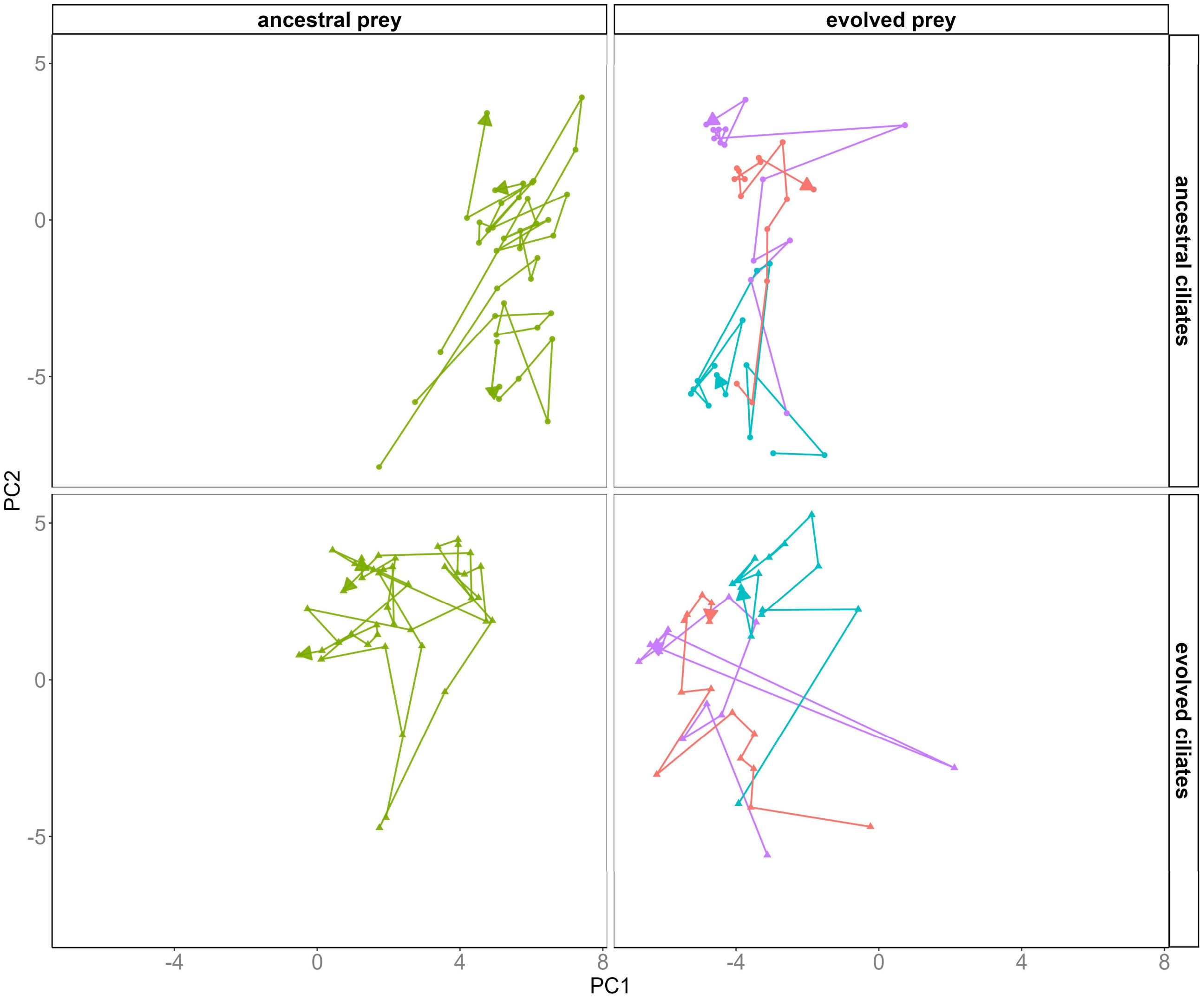

Figures S2 Community trajectories with predators. Community composition from 16s rRNA amplicon sequencing was analysed by PCA on the centred log ratio transform of species relative abundance (Aitchisons distance). Trajectories of communities from individual microcosms are represented in the two scaling dimensions. Direction of the trajectories is indicated by the arrow pointing to the last sampling at day 60. Shapes indicate the evolutionary history of the predator: circles = naive predators, triangles = evolved predators. Colours represent evolutionary history of the prey: green = ancestral prey, violet/cyan/red = evolved prey lineages A/B/C. Trajectories are directional along PC1 and trajectories of ancestral and evolved prey communities are separated by PC2.

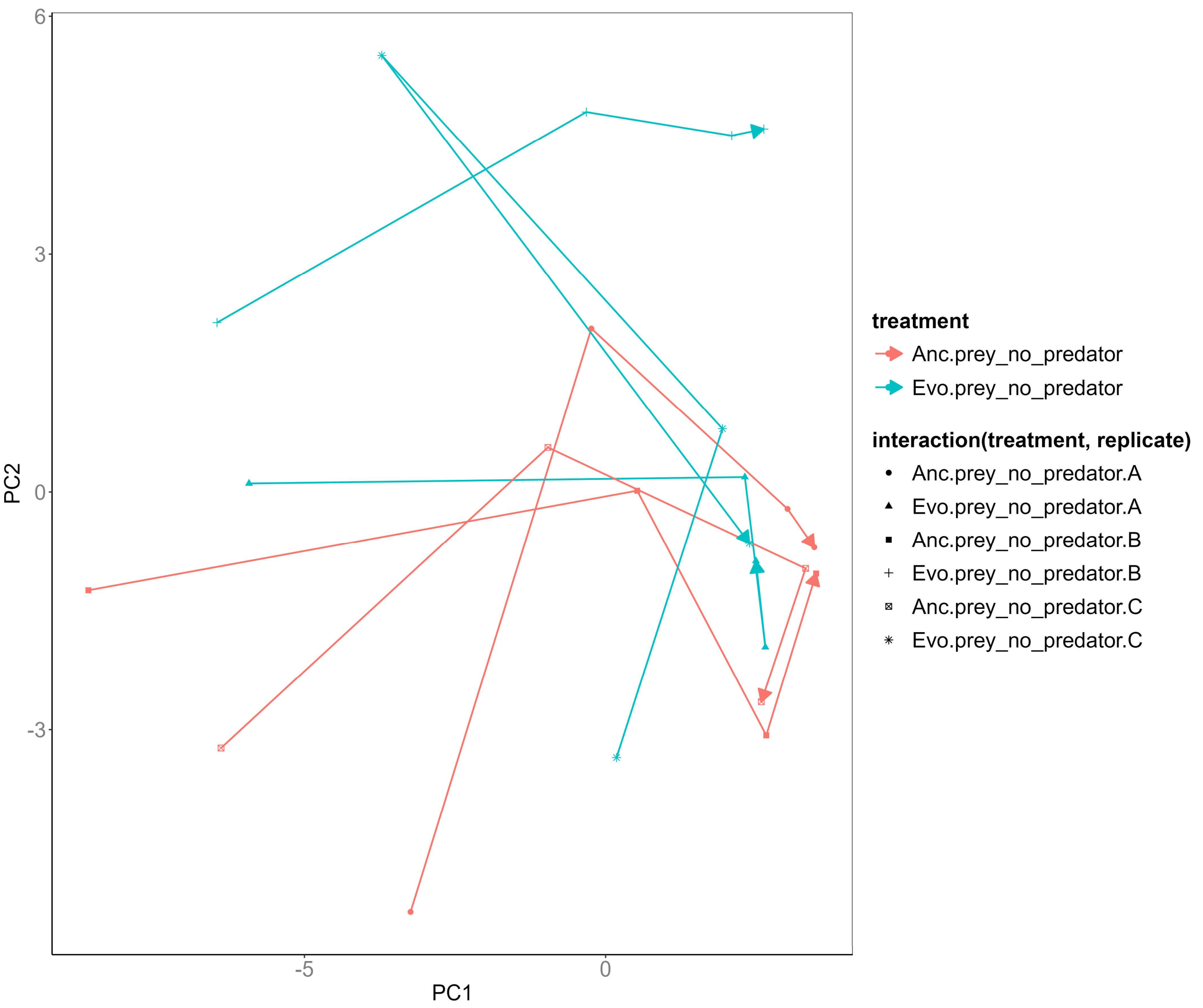

Figure S3 Community Trajectories without predators. Other than in microcosms with predators, microcosms without predators were sampled at days 12, 28, 44,60 and thus separately analysed. These communities were likewise analysed by PCA (Aitchison distance). Community trajectories are here displayed on the two principal axes of this ordination. The arrow points to the day 60 community. Irrespective of prey evolutionary history five of the six communities seem to converge to relatively similar compositions. Colour: red=ANC prey, blue=EVO prey. Shapes distinguish the individual trajectories/microcosms.

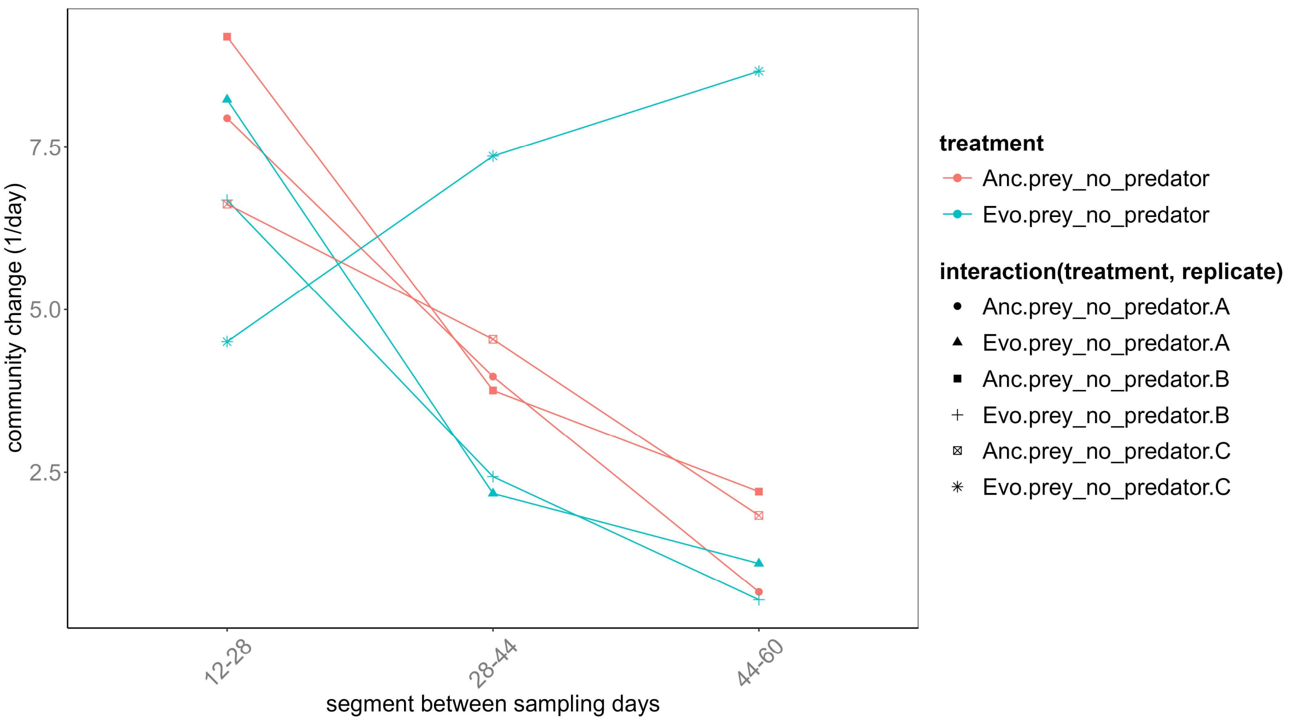

Figure S4 Dynamics of species sorting without predators. Analogous to the communities in predator containing microcosms, trajectories from communities in predator free microcosms were analysed by estimating the rate of community change via the length of segments between consecutive sampling days. Irrespective of prey evolutionary history, community change dropped drastically during the experiment in five of the six communities. This indicates that communities reached a relatively stable composition at the end of the experiment. Colour: red=ANC prey, blue=EVO prey. Shapes distinguish the individual trajectories/microcosms.

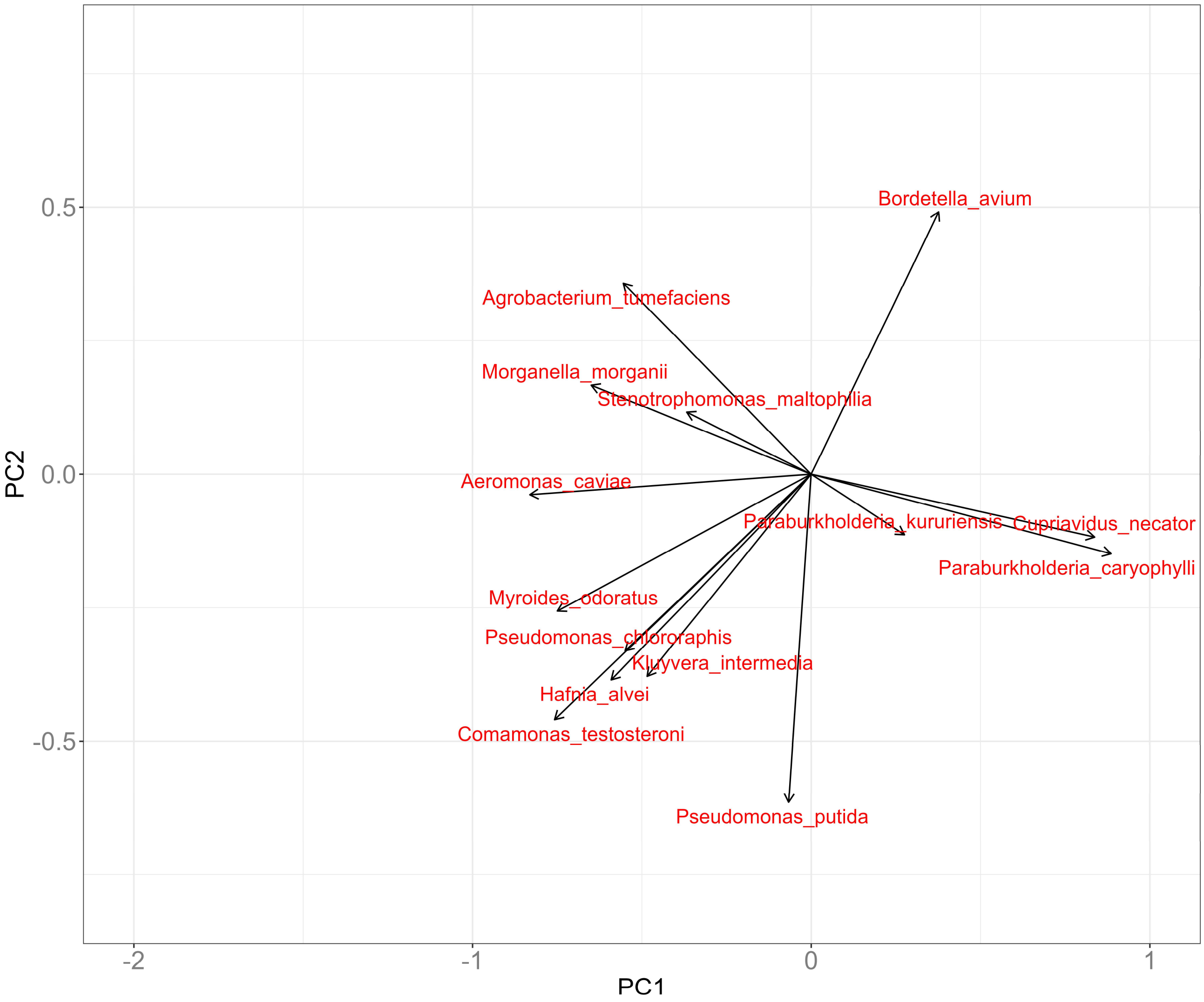

Figure S5 Species impact. Community composition from 16s rRNA amplicon sequencing was analysed by PCoA based on Aitchison distance (Figure S2). The envfit function (R package vegan) was used to project specie's abundances as environmental vectors onto the ordination via linear regression. Species with significant coefficients ($p<0.001$) were considered to have a significant impact on composition of communities and their coefficients were plotted as vectors in the ordination. Arrows starting from the origin point toward increasing abundance of the corresponding species and the length of arrows indicates the relative impact on community composition.

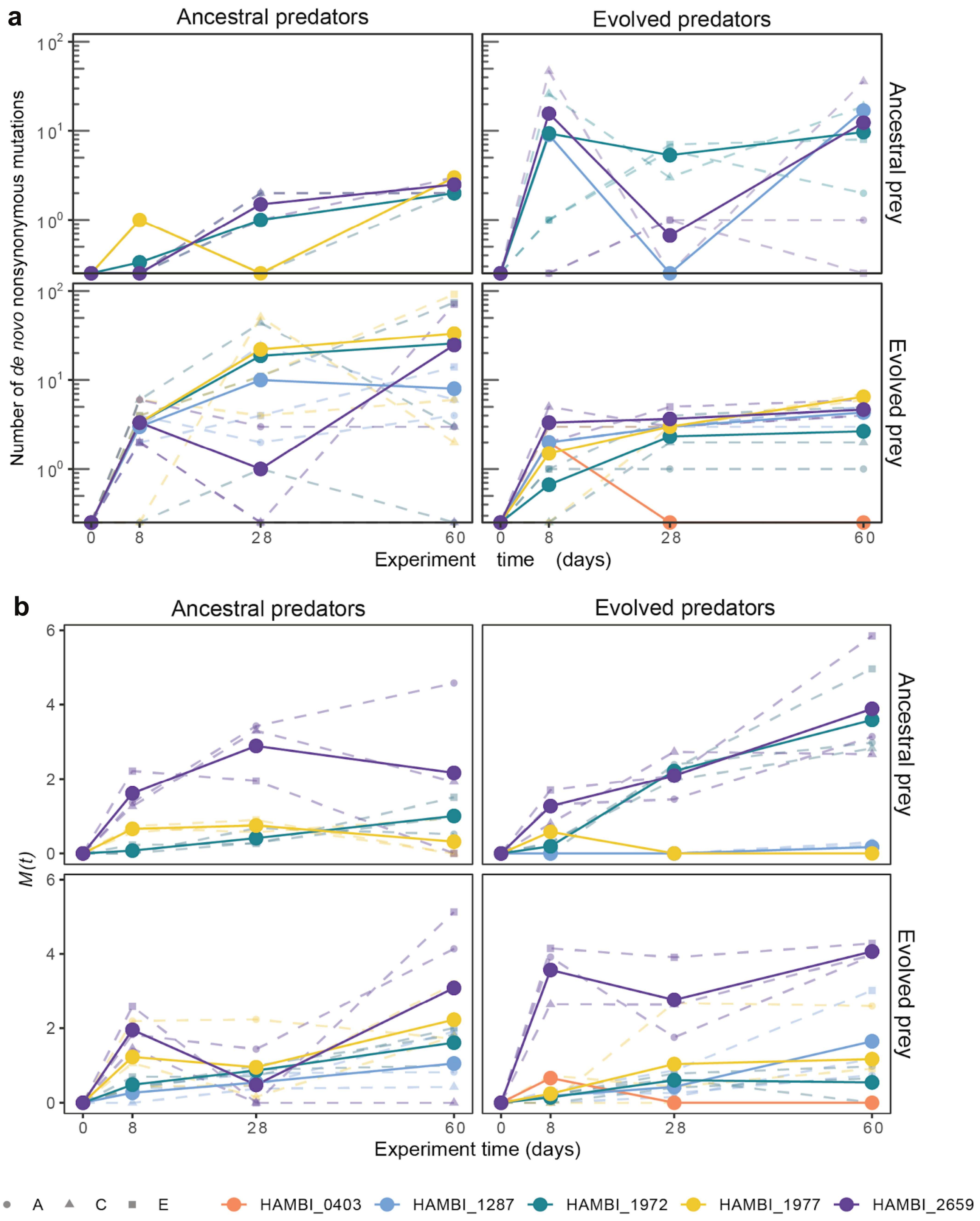

Figure S6 De novo mutational trajectories saturate with time. a) Trajectories of the total number of non-zero mutations detected at each time point for de novo (i.e., excludes standing genetic variation in the evolved populations) amino acid altering variants in HAMBI genomes with metagenomic coverage > 5X in at least one time point. **b**) Cumulative mutation trajectories (*M(t)*) of the same variants. Transparent points/lines represent individual replicates, while opaque points/lines represent the mean over the three replicates.

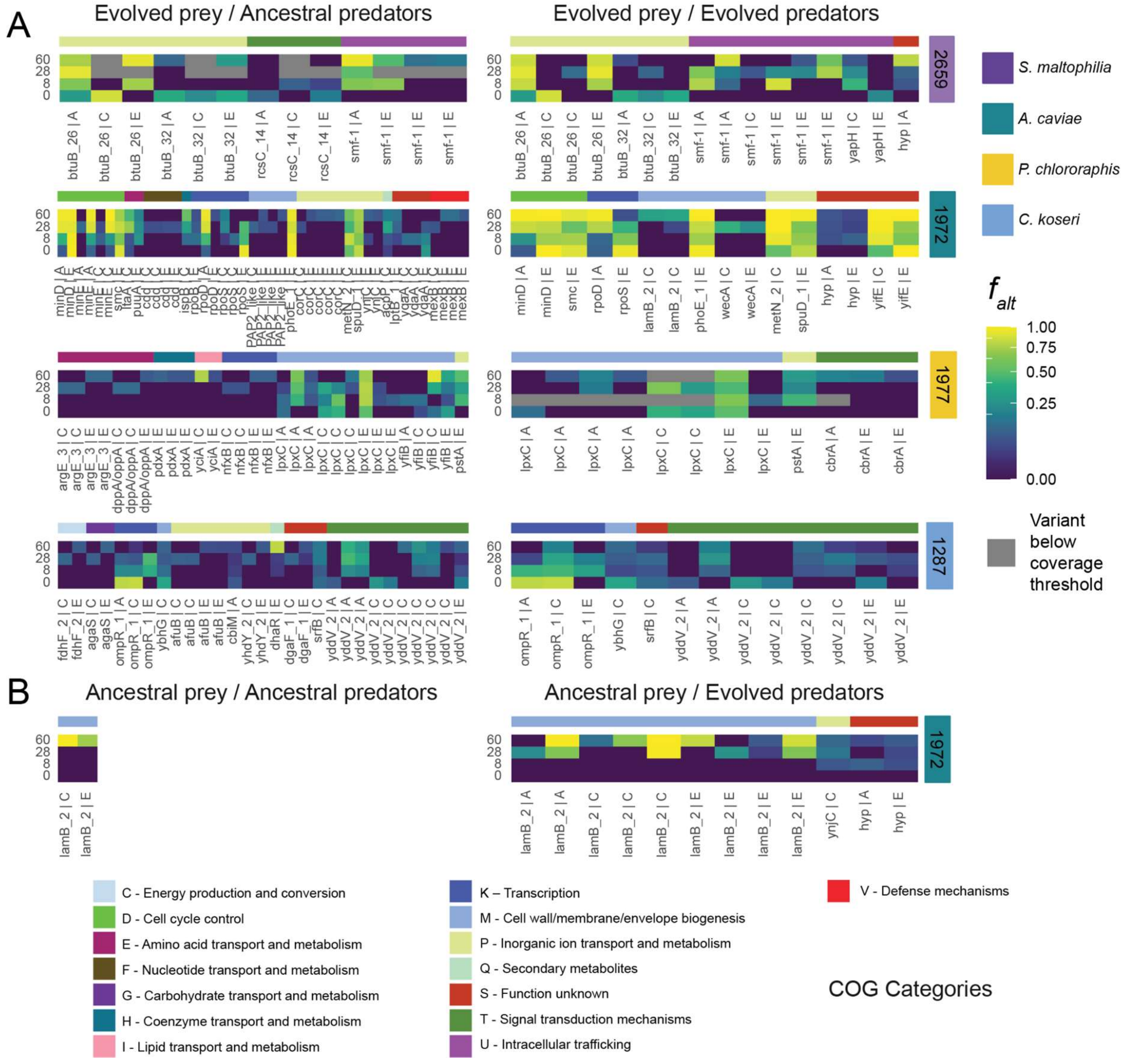

Figure S7 Heatmap presentation of parallel mutational trajectories. Mutational trajectories for significantly parallel genes identified in species (rows/colors) in conditions of A) evolved prey and B) ancestral prey match-mismatch with ancestral (left) and evolved (right) predators. Frequencies of individual mutations (heat map cell color) are organized by gene and experimental replicate. Genes are color-coded according to their COG functional category (color bar above heatmaps).

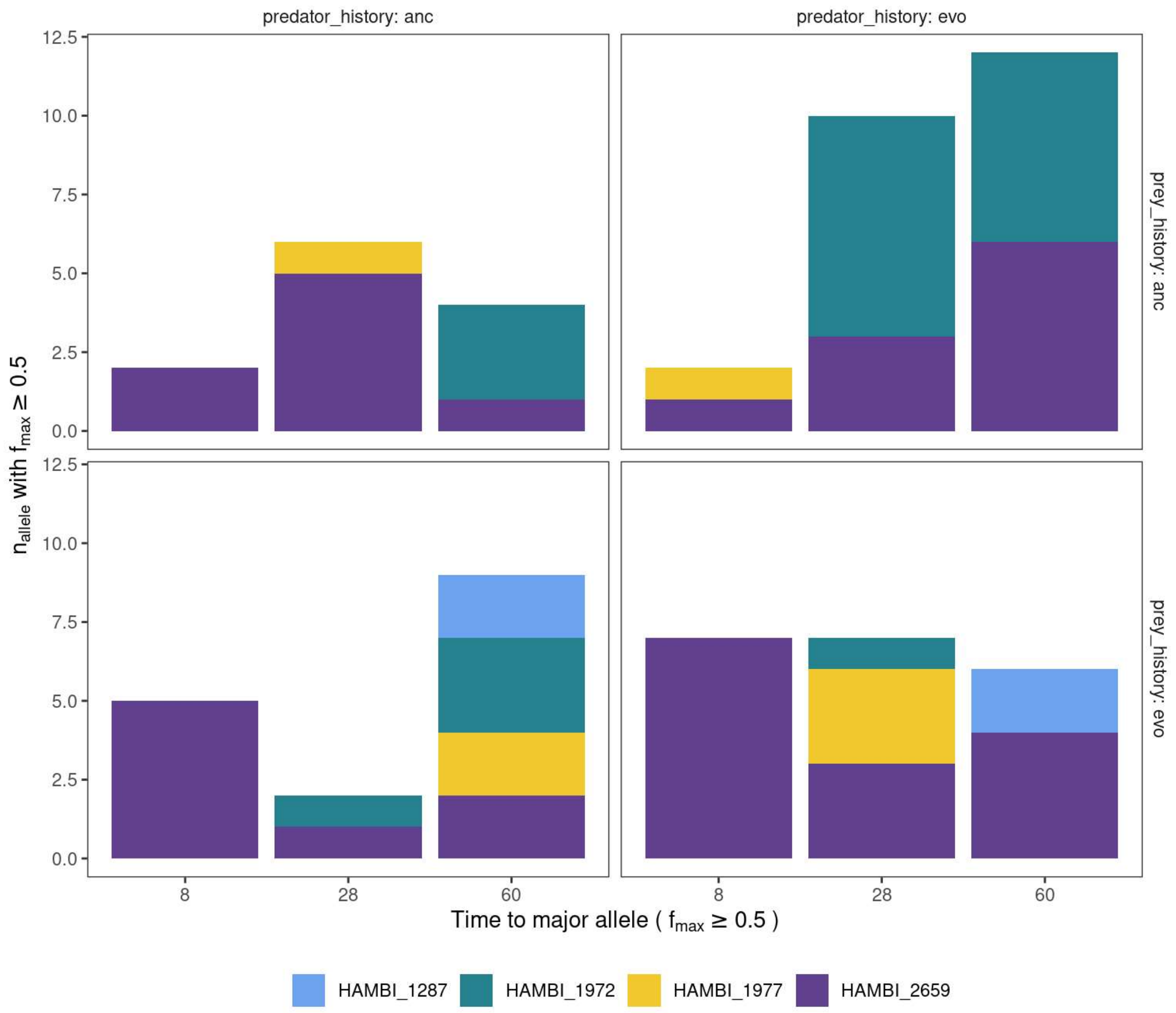

Figure S8 Time to major allele status. Count of alternative alleles with $f_{max} \geq 0.5$ plotted against the time (days) when the alternative allele first reaches major allele status ($f_{max} \geq 0.5$). Colors represent the proportion of alleles from different species. The plot includes only amino acid changing de novo variants (i.e., excludes standing genetic variation from the evolved bacterial populations).

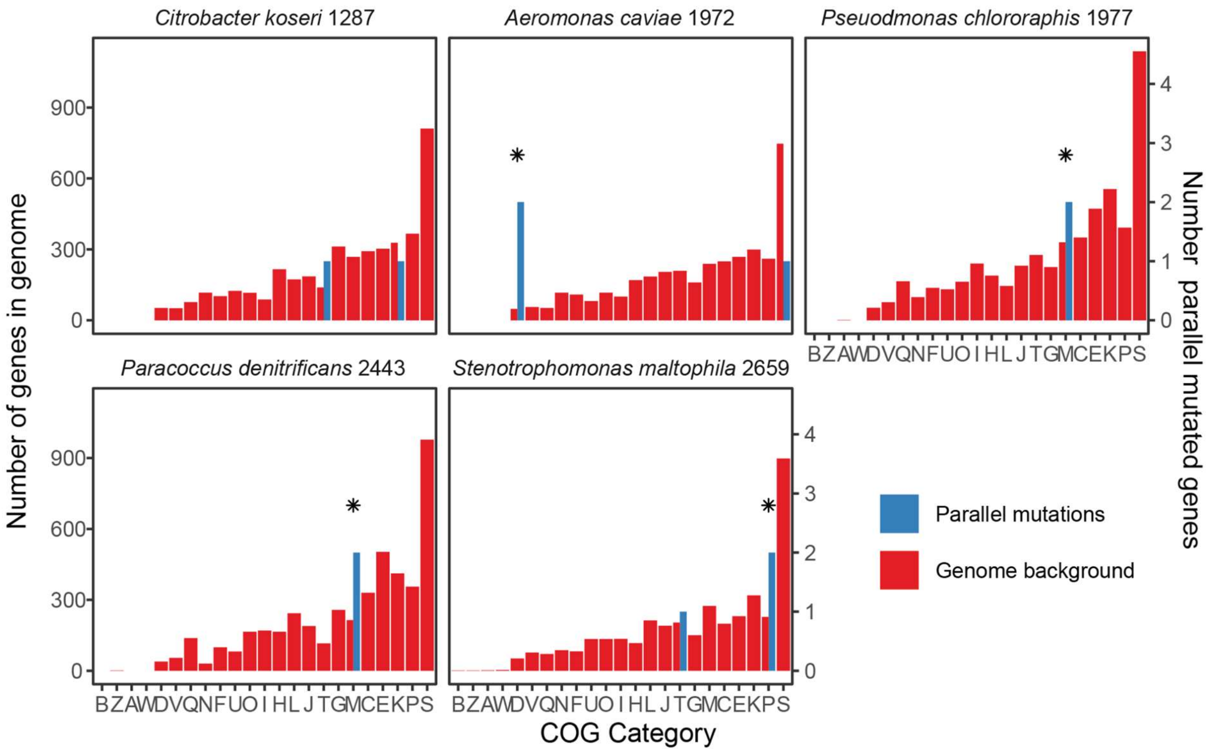

Figure S9 Functional enrichments in parallel mutated genes from starting evolved populations. Fraction of genes in each Cluster of Orthologous Groups (COG) category for all genes with a COG annotation (red bars, left y-axis) and only genes exhibiting significant genomic parallelism (blue bars, right y-axis) for the four most abundant genomes in the metagenomes (1287, 1972, 1977, and 2659) and *Paracococus denitrificans* 2443 which had significant clustering of parallel mutations (hypergeometric test, q-value for positive false discovery rate control). COG categories "D - Cell cycle control, cell division, chromosome partitioning" for *A. caviae* 1972 (*q*-value = 0.006), "M - Cell wall/membrane/envelope biogenesis" for *P. chlororaphis* 1977 (*q*-value = 0.015) and *P. denitrificans* 2443 (*q*-value = 0.013), and "P - Inorganic ion transport and metabolism" for *S. maltophilia* 2659 (*q*-value = 0.031) are significantly enriched (denoted with *) in the parallel mutations compared to the rest of the genome for these species.

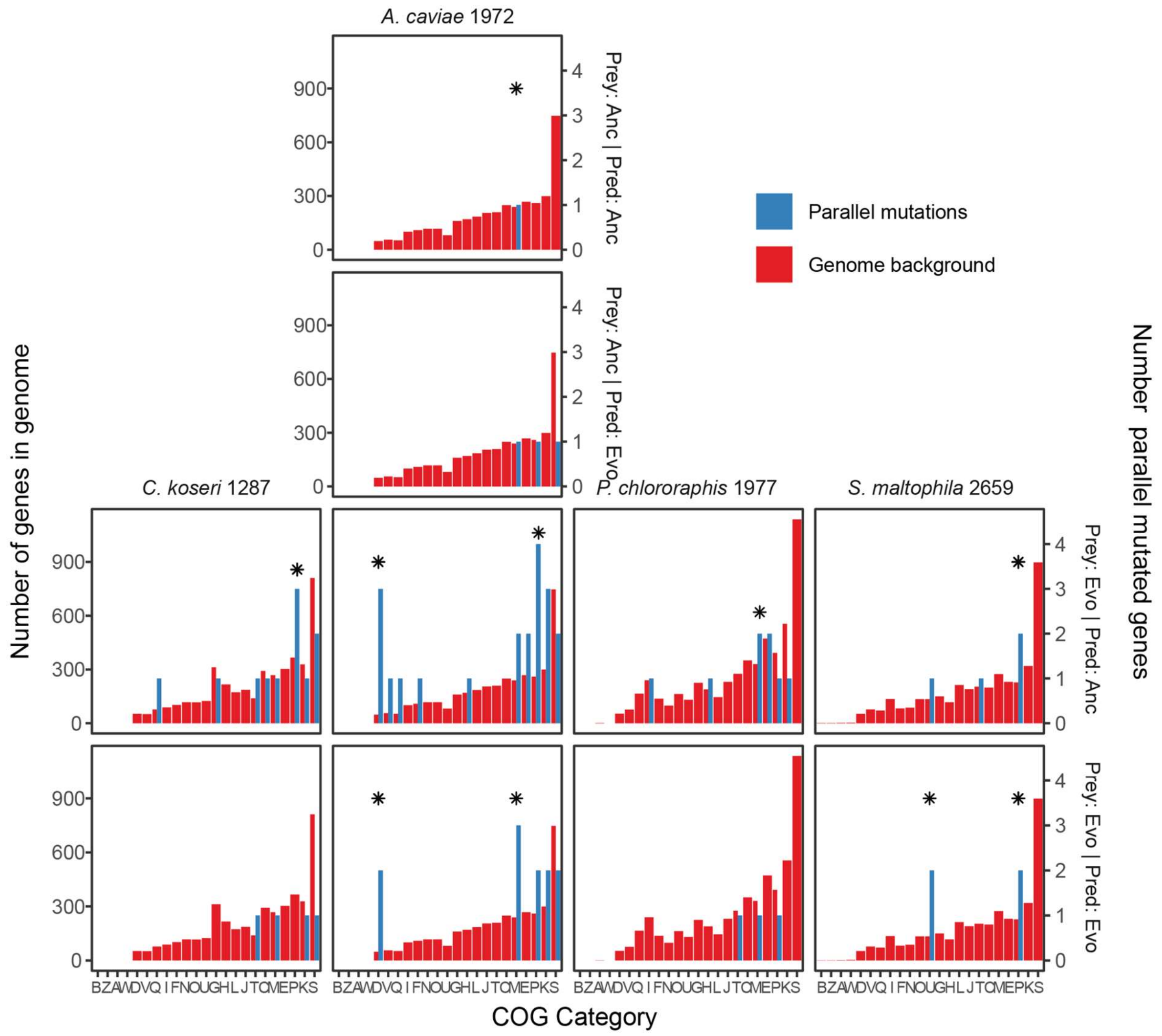

Figure S10 Functional enrichments in parallel mutated genes during the community experiment. Fraction of genes in each Cluster of Orthologous Groups (COG) category for genes with a COG annotation (red bars, left y-axis) and only genes exhibiting significant genomic parallelism (blue bars, right y-axis) for the four most abundant HAMBI species detected in the metagenomes. COG categories for species by match-mismatch combinations are denoted with * if they are significantly enriched in parallel mutations relative to the genomic background (hypergeometric test, *q*-value for positive false discovery rate control). *C. koseri* 1287, COG P, Prey: Evo | Pred: Anc *q*-value = 0.068; *A. caviae* 1972, COG D, Prey: Evo | Pred: Anc *q*-value = 0.018, Prey: Evo | Pred: Evo *q*-value = 0.026, COG M, Prey: Anc | Pred: Anc *q*-value = 0.068, Prey: Evo | Pred: Evo *q*-value = 0.046, COG P, Prey: Evo | Pred: Anc *q*-value = 0.063; *P. chlororaphis* 1977, COG M, Prey: Evo | Pred: Anc *q*-value = 0.075; *S. maltophilia* 2659, COG U, Prey: Evo | Pred: Evo *q*-value = 0.026, COG P, Prey: Evo | Pred: Anc *q*-value = 0.036, Prey: Evo | Pred: Evo *q*-value = 0.036.

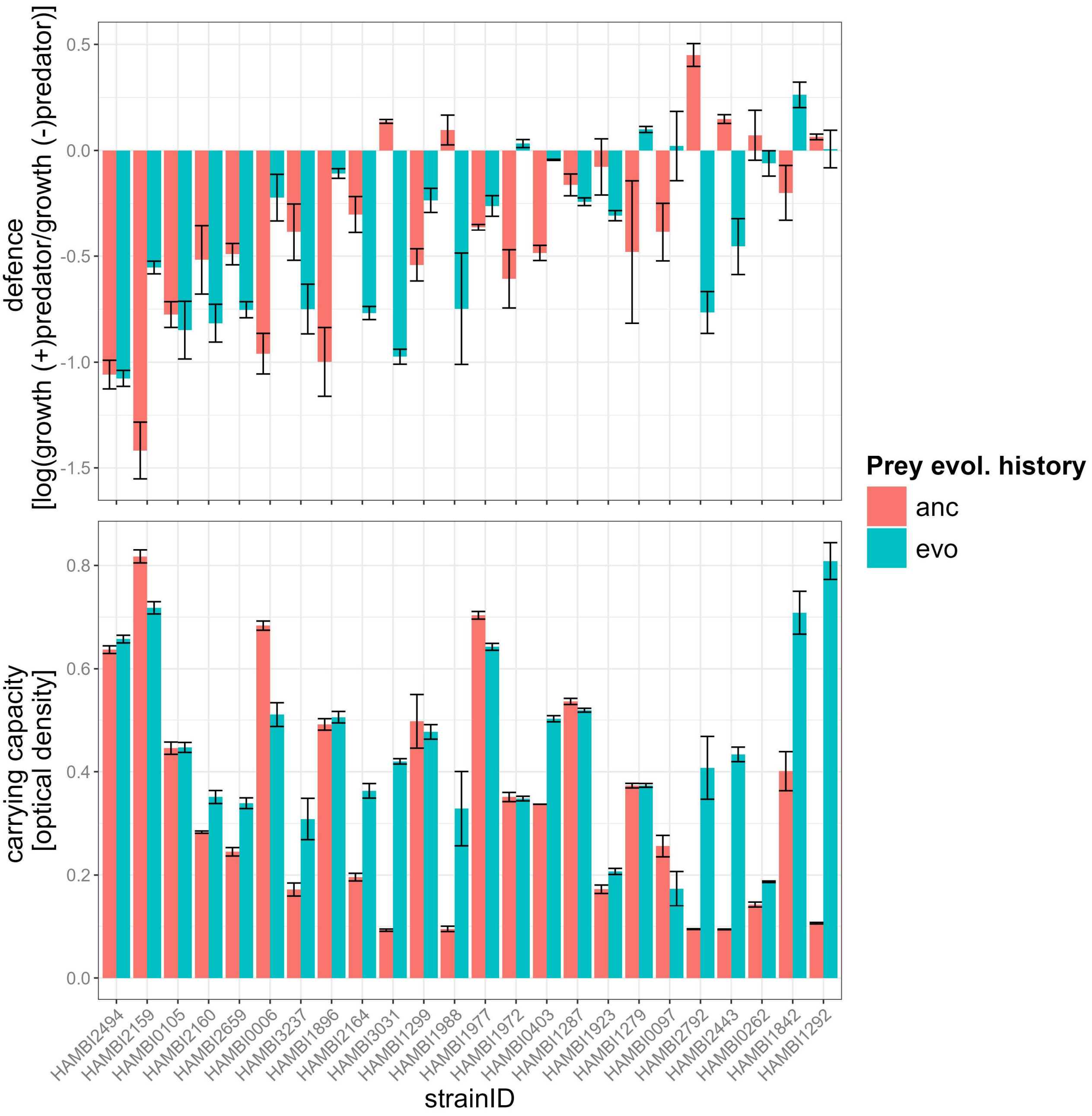

Figure S11 Initial traits. Carrying capacity and anti-predator defence of the 24 ancestral HAMBI species (ANC) and the corresponding 24 evolved populations of those species (EVO). Shown are median values of 8 randomly picked clones from each species and evolutionary history. Error bars represent the median absolute deviation (MAD). The species on the x-axis are sorted according to ascending defence values. The ancestral species responded in two ways to the 100-day co-culture with predators: 10 of 24 species evolved higher defence than their ancestors, 17 of 24 species evolved higher carrying capacity than their ancestors and only 4 species were able to improve both traits.

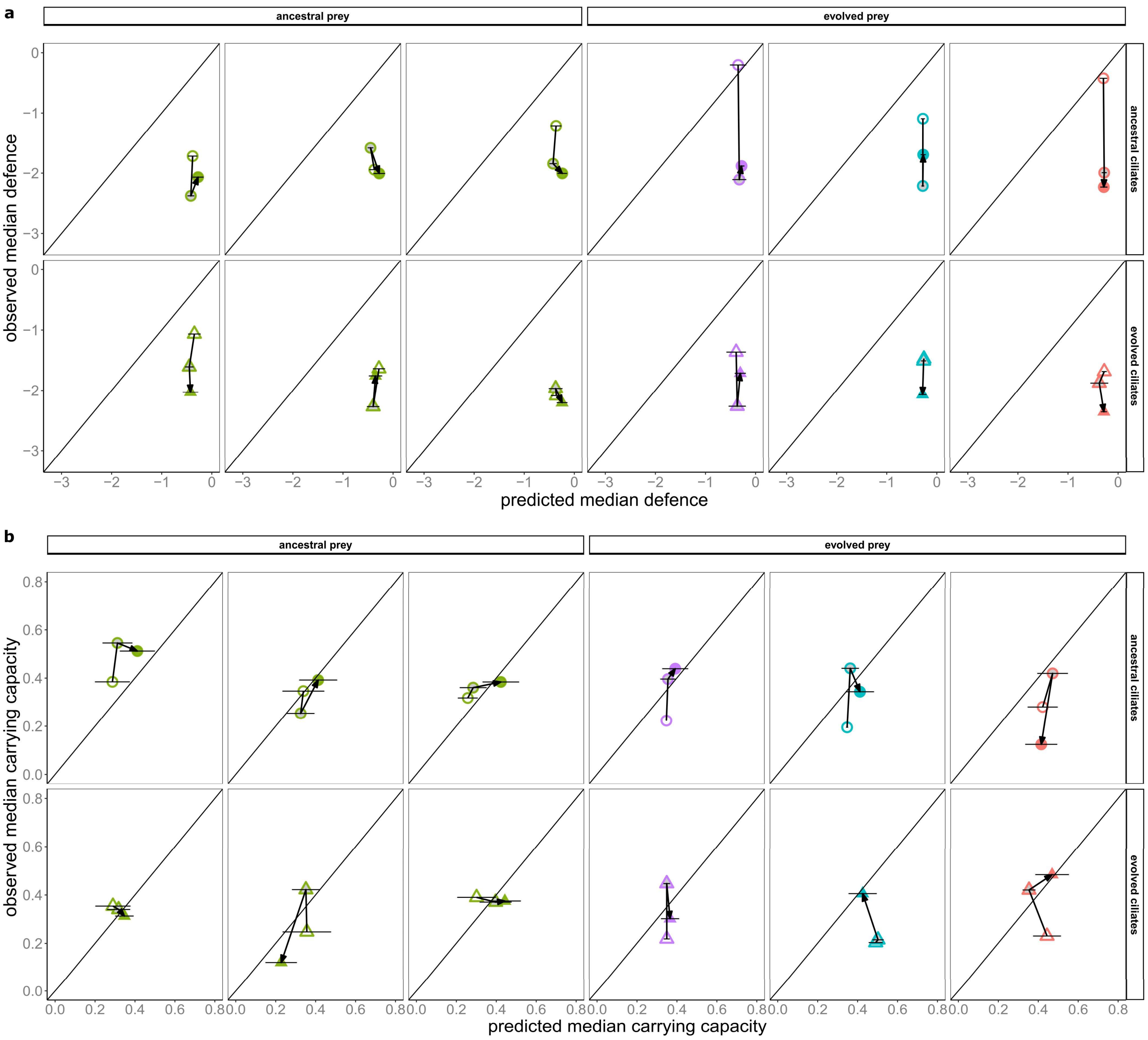

Figure S12 Contribution of species sorting and evolution to phenotypic change. Data from the analysis in Fig. 5 is alternatively displayed for defence (**a**) and carrying capacity (**b**). The average median trait value of 100 samples predicted by species sorting is plotted against the median observed trait value for each microcosm and sampling day. Error bars represent the standard deviation of the 100 median trait values predicted by species sorting. The diagonal line of slope 1 represents cases where predicted and observed median traits align. The arrows point from data on day 8 (open shapes) to data on days 28 (grey filled shapes) and 60 (filled shapes). Data point shape indicates evolutionary history of the predator: circles = ancestral predators, triangles = evolved predators. Colours represent evolutionary history of the prey: green = ancestral prey, violet/cyan/red = evolved prey lineages A/B/C.

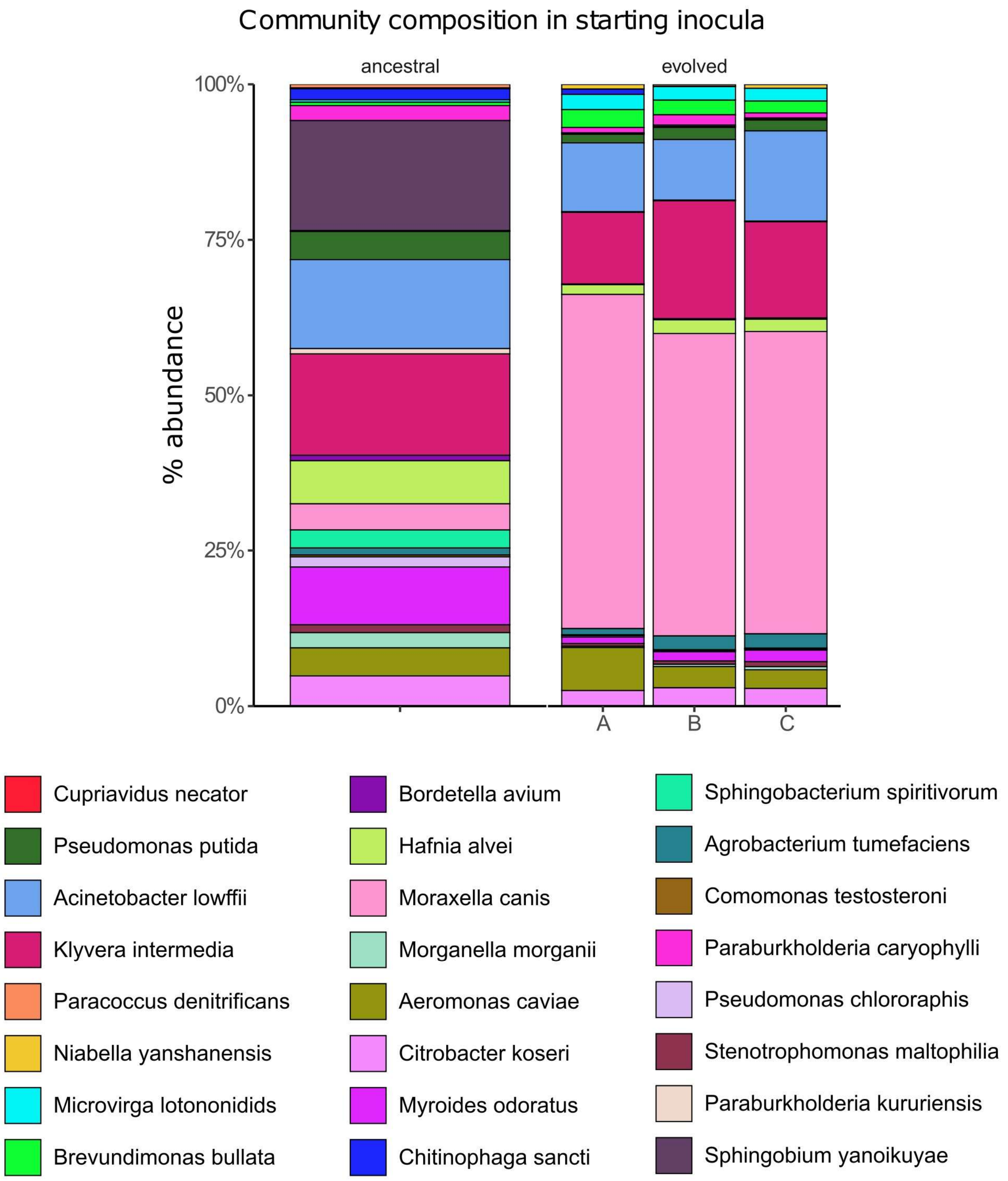

Figure S13 Starting inocula. 16s rRNA amplicon sequencing of the ancestral community inoculum (left) and the evolved community inocula (right) where all HAMBI species were added at approximately equal proportions. The single ancestral inoculum was used to start all ancestral prey microcosms. The evolved inocula were assembled from one of three evolved populations per species. Each evolved inoculum was used to start one microcosm with evolved prey (A, B, C) under the three predator conditions (ancestral, evolved, predator free).

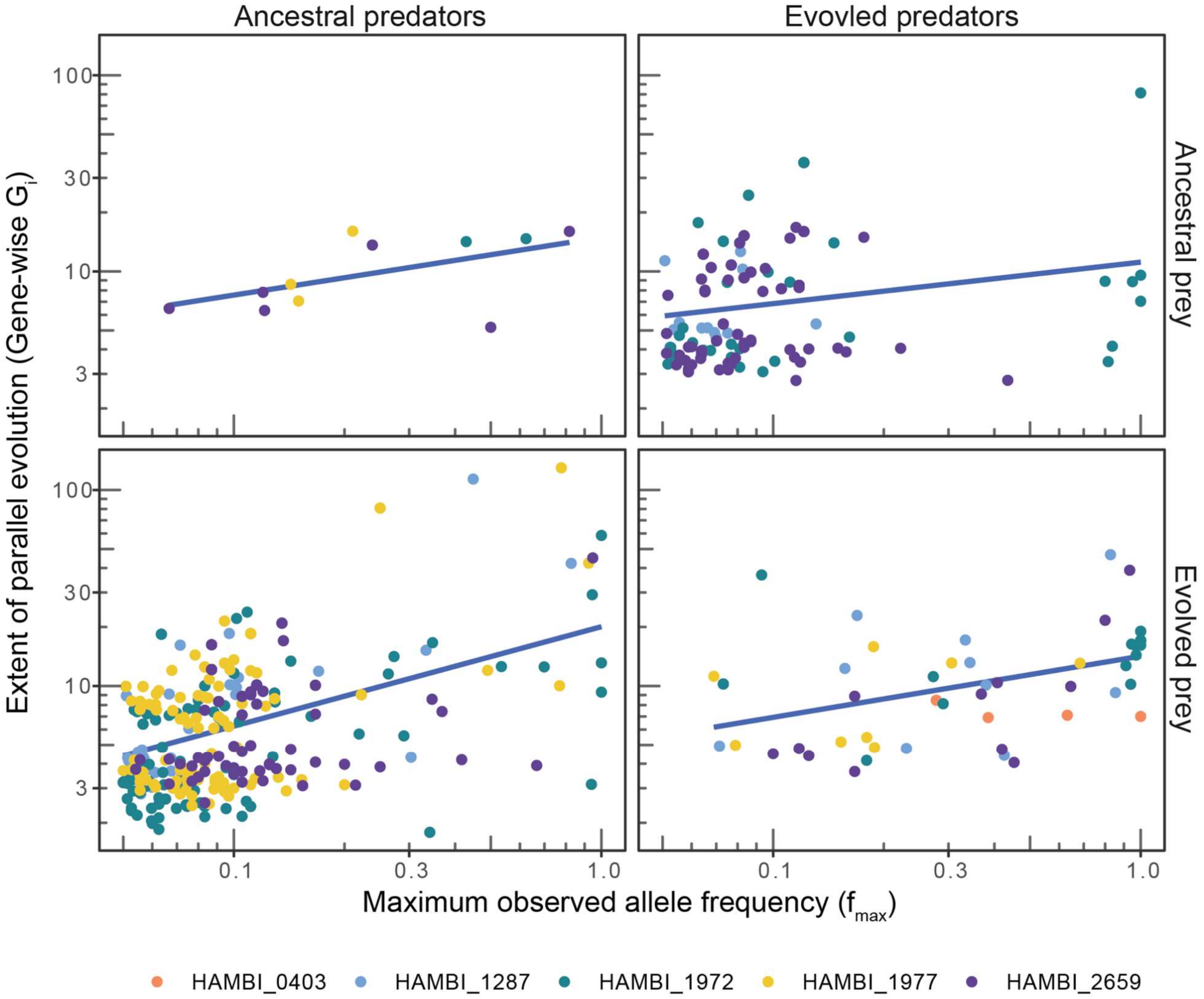

Figure S14 Higher allele frequencies in parallel mutated genes. Relationship between parallel evolution at the gene level (G_i, vertical axis) and the maximum observed allele frequency of mutations in each gene (horizontal axis). Points are colored by species. Blue lines show a linear regression for each experimental match/mismatch combination (over species). Overall, we find a clear positive relationship between the gene-wise extent of parallel evolution (G_i) and the maximum observed allele frequency for that gene in 3/4 treatment combinations ($P < 0.05$). We also find a positive relationship for the evolved prey/ancestral predator combination, but the significance of this relationship is unclear (P value = 0.07).

Supporting Tables

Phenotypic change

Table S1. Defence - Kruskal-Wallis Test day 8 vs day 60 trait distribution. P-values for 12 individual Kruskal-Wallis (stats::kruskall.test R) tests to detect significant differences between defence distributions on day 8 and day 60 for each replicate microcosm. Corrected p-values were derived by performing Benjamin-Hochberg procedure for multiple testing. We took $p < 0.05$ as significance threshold.

prey	predator	replicate	p.value	rank_sum_statistic	Df	p.value_corr
Ancestral Bacteria	Ancestral Ciliates	A	0.015	5.886	1	0.026
Ancestral Bacteria	Ancestral Ciliates	B	0.136	2.219	1	0.182
Ancestral Bacteria	Ancestral Ciliates	C	0.006	7.537	1	0.014
Ancestral Bacteria	Evolved Ciliates	A	0.007	7.238	1	0.014
Ancestral Bacteria	Evolved Ciliates	B	0.893	0.018	1	0.893
Ancestral Bacteria	Evolved Ciliates	C	0.207	1.596	1	0.248
Evolved Bacteria	Ancestral Ciliates	A	0.000	32.188	1	0.000
Evolved Bacteria	Ancestral Ciliates	B	0.000	23.772	1	0.000
Evolved Bacteria	Ancestral Ciliates	C	0.003	8.571	1	0.010
Evolved Bacteria	Evolved Ciliates	A	0.360	0.838	1	0.393
Evolved Bacteria	Evolved Ciliates	B	0.019	5.467	1	0.029
Evolved Bacteria	Evolved Ciliates	C	0.000	14.931	1	0.000

Table S2. Carrying capacity - Kruskal-Wallis Test day 8 vs day 60 trait distribution. P-values for 12 individual Kruskal-Wallis (stats::kruskal.test R) tests to detect significant differences between growth capacity distributions on day 8 and day 60 for each replicate microcosm. Corrected p-values were derived by performing Benjamin-Hochberg procedure for multiple testing. We took $p < 0.05$ as significance threshold.

prey	predator	replicate	p.value	rank_sum_statistic	Df	p.value_corr
Ancestral Bacteria	Ancestral Ciliates	A	0.053	3.757	1	0.090
Ancestral Bacteria	Ancestral Ciliates	B	0.080	3.073	1	0.119
Ancestral Bacteria	Ancestral Ciliates	C	0.006	7.409	1	0.016
Ancestral Bacteria	Evolved Ciliates	A	0.821	0.051	1	0.895
Ancestral Bacteria	Evolved Ciliates	B	0.014	6.024	1	0.028
Ancestral Bacteria	Evolved Ciliates	C	0.992	0.000	1	0.992
Evolved Bacteria	Ancestral Ciliates	A	0.000	29.637	1	0.000
Evolved Bacteria	Ancestral Ciliates	B	0.000	21.337	1	0.000
Evolved Bacteria	Ancestral Ciliates	C	0.090	2.868	1	0.120
Evolved Bacteria	Evolved Ciliates	A	0.194	1.688	1	0.233
Evolved Bacteria	Evolved Ciliates	B	0.005	7.981	1	0.014
Evolved Bacteria	Evolved Ciliates	C	0.000	19.202	1	0.000

Community composition

Table S3a. PCoA global - PERMANOVA. The PERMANOVA was performed on the species relative abundance derived from 16s rRNA amplicon-sequencing in all microcosms. Microcosms without predators were sampled at lower frequency and therefore contribute with fewer samples. Computation was performed via vegan::adonis2 using Aitchison distance as dissimilarity metric. Formula: aitchison_distance ~ prey_history + predator_history P-values < 0.05 were considered significant.

	**Df**	**SumOfSqs**	**R2**	**F**	**Pr(>F)**
prey_history	1	2380.342	0.230373	77.63567	0.000999
predator_history	2	1820.123	0.176154	29.68196	0.000999
Residual	200	6132.084	0.593473	NA	NA
Total	203	10332.549	1	NA	NA

Table S3b. Permutation test for prey history. We checked for homogeneity of variance based on grouping by "prey_history" (betadisper R package vegan) and then performed a permutation test (permutest R package vegan). P-values < 0.05 were considered significant.

**Response:Distances**	**Df**	**Sum Sq**	**Mean Sq**	**F**	**N.Perm**	**Pr(>F)**
Groups	1	38.61	38.613	9.4672	999	0.006
Residuals	202	823.88	4.079			

Table S3c. Permutation test for prey history. We checked for homogeneity of variance based on grouping by "predator_history" (betadisper R package vegan) and then performed a permutation test (permutest R package vegan). P-values < 0.05 were considered significant.

**Response:Distances**	**Df**	**Sum Sq**	**Mean Sq**	**F**	**N.Perm**	**Pr(>F)**
Groups	**2**	**136.53**	**68.263**	**34.83**	**999**	**0.001**
Residuals	**201**	**393.94**	**1.96**			

Table S4. PERMANOVA on community trajectories in microcosms with predators. The PERMANOVA was performed on the species relative abundance derived from 16s rRNA amplicon-sequencing in the predator containing microcosms. Computation was performed via vegan::adonis2 using Euclidean distance based on the mean-centred and log-transformed abundances (Aitchison distance). Replicate microcosms were considered as experimental units (plots) and permutation within plots was handled as time series to account for the repeated sampling of microcosms over time. Formula: species ~ prey_history * predator_history * time_days. We corrected for temporal pseudoreplication by setting permutation within microcosms (experimental units) to type time series. P-values < 0.05 were considered significant. Multivariate dispersion was tested via vegan::betadisper followed by ANOVA which gave a non-significant result (P = 0.9994, F = 0.444, Df = 55) showing there was no over-dispersion between groups and validating PERMANOVA results.

variable	Df	SumOfSqs	R2	F	Pr(>F)
character	numeric	numeric	numeric	numeric	numeric
prey_history	1	2,530.457	0.315	105.246	0.001
predator_history	1	609.565	0.076	25.353	0.089
time_days	1	340.998	0.042	14.183	0.001
prey_history:predator_history	1	528.202	0.066	21.969	0.128
prey_history:time_days	1	109.580	0.014	4.558	0.001
predator_history:time_days	1	29.297	0.004	1.219	0.261
prey_history:predator_history:time_days	1	28.660	0.004	1.192	0.261
Residual	160	3,846.928	0.479		
Total	167	8,023.689	1.000		

n: 9

Table S5. Directionality of trajectories. Given is the directionality of community trajectories within the first two dimensions of the PCoA. The directionality was calculated via the trajectoryDirectionality function of the R package ecotraj. The directionality is given as a value between 0 and 1, where zero indicates no directionality and 1 complete directionality. All trajectories are moderately directional.

**Directionality of trajectories within PCoA**	
**sites**	**traj_direct**
Anc.prey_Anc.predator_A	0.328
Anc.prey_Anc.predator_B	0.377
Anc.prey_Anc.predator_C	0.472
Anc.prey_Evo.predator_A	0.451
Anc.prey_Evo.predator_B	0.464
Anc.prey_Evo.predator_C	0.405
Evo.prey_Anc.predator_A	0.558
Evo.prey_Anc.predator_B	0.313
Evo.prey_Anc.predator_C	0.540
Evo.prey_Evo.predator_A	0.384
Evo.prey_Evo.predator_B	0.388
Evo.prey_Evo.predator_C	0.618

Genomic evolution

Table S6. List of parallel mutated genes. See supplementary excel file (Table S6.xlsx).

Contributions of species sorting and evolution

Table S7. Regression - Deviation ratio defence. Coefficients for regressions over resampled deviation ratios (log(observed median trait/predicted median trait)) along the three sampling days were determined by fitting a linear model (stats::lm) of the formula: ratio_predicted_observed_df ~ Replicate_unique/time_days -1. The model yielded an intercept and slope for each replicate microcosm. P-values < 0.05 were considered significant.

prey_history	predator_history	replicate	Replicate_unique	Estimate	Std. Error	t value	Pr(>|t|)	type
Anc_prey	Anc_predator	A	1	1.45041	0.04154	34.92	4.019e-230	intercept
Anc_prey	Anc_predator	B	2	1.36858	0.04154	32.95	3.819e-208	intercept
Anc_prey	Anc_predator	C	3	1.11035	0.04154	26.73	1.133e-143	intercept
Evo_prey	Anc_predator	A	4	-0.14135	0.04154	-3.40	6.743e-04	intercept
Evo_prey	Anc_predator	B	5	1.51998	0.04154	36.59	2.716e-249	intercept
Evo_prey	Anc_predator	C	6	0.54703	0.04154	13.17	1.031e-38	intercept
Anc_prey	Evo_predator	A	7	1.09881	0.04154	26.45	5.476e-141	intercept
Anc_prey	Evo_predator	B	8	1.89501	0.04154	45.62	0.000e+00	intercept
Anc_prey	Evo_predator	C	9	1.74287	0.04154	41.96	3.173e-313	intercept
Evo_prey	Evo_predator	A	10	1.54010	0.04154	37.08	6.483e-255	intercept
Evo_prey	Evo_predator	B	11	1.69303	0.04154	40.76	1.220e-298	intercept
Evo_prey	Evo_predator	C	12	1.69597	0.04154	40.83	1.707e-299	intercept
Anc_prey	Anc_predator	A	1	0.01012	0.00108	9.39	1.081e-20	slope
Anc_prey	Anc_predator	B	2	0.00953	0.00108	8.83	1.582e-18	slope
Anc_prey	Anc_predator	C	3	0.01681	0.00108	15.58	4.957e-53	slope
Evo_prey	Anc_predator	A	4	0.04043	0.00108	37.48	1.345e-259	slope
Evo_prey	Anc_predator	B	5	0.00773	0.00108	7.16	9.435e-13	slope
Evo_prey	Anc_predator	C	6	0.02929	0.00108	27.15	9.143e-148	slope
Anc_prey	Evo_predator	A	7	0.00895	0.00108	8.30	1.449e-16	slope
Anc_prey	Evo_predator	B	8	-0.00140	0.00108	-1.29	1.956e-01	slope
Anc_prey	Evo_predator	C	9	0.00604	0.00108	5.60	2.259e-08	slope
Evo_prey	Evo_predator	A	10	0.00480	0.00108	4.45	8.789e-06	slope
Evo_prey	Evo_predator	B	11	0.00438	0.00108	4.06	4.999e-05	slope
Evo_prey	Evo_predator	C	12	0.00621	0.00108	5.75	9.531e-09	slope

Table S8. Regression - Deviation ratio carrying capacity. Coefficients for regressions over resampled deviation ratios (log(observed median trait/predicted median trait)) along the three sampling days were determined by fitting a linear model (stats::lm) of the formula: ratio_predicted_observed_growth ~ Replicate_unique/time_days -1. The model yielded an intercept and slope for each replicate microcosm. P-values < 0.05 were considered significant.

prey_history	predator_history	replicate	Replicate_unique	Estimate	Std. Error	t value	Pr(>|t|)	type
Anc_prey	Anc_predator	A	1	0.46766	0.029128	16.055	4.581e-56	intercept
Anc_prey	Anc_predator	B	2	0.04157	0.029128	1.427	1.536e-01	intercept
Anc_prey	Anc_predator	C	3	0.31663	0.029128	10.870	4.217e-27	intercept
Evo_prey	Anc_predator	A	4	-0.39403	0.029128	-13.528	1.061e-40	intercept
Evo_prey	Anc_predator	B	5	-0.37815	0.029128	-12.982	1.080e-37	intercept
Evo_prey	Anc_predator	C	6	-0.01105	0.029128	-0.379	7.045e-01	intercept
Anc_prey	Evo_predator	A	7	0.27095	0.029128	9.302	2.336e-20	intercept
Anc_prey	Evo_predator	B	8	0.04175	0.029128	1.433	1.519e-01	intercept
Anc_prey	Evo_predator	C	9	0.23571	0.029128	8.092	7.962e-16	intercept
Evo_prey	Evo_predator	A	10	-0.25414	0.029128	-8.725	4.015e-18	intercept
Evo_prey	Evo_predator	B	11	-1.11688	0.029128	-38.344	9.098e-270	intercept
Evo_prey	Evo_predator	C	12	-0.53228	0.029128	-18.274	2.167e-71	intercept
Anc_prey	Anc_predator	A	1	-0.00253	0.000756	-3.344	8.341e-04	slope
Anc_prey	Anc_predator	B	2	-0.00261	0.000756	-3.453	5.602e-04	slope
Anc_prey	Anc_predator	C	3	-0.00576	0.000756	-7.618	3.267e-14	slope
Evo_prey	Anc_predator	A	4	0.01047	0.000756	13.841	1.769e-42	slope
Evo_prey	Anc_predator	B	5	0.00632	0.000756	8.361	8.819e-17	slope
Evo_prey	Anc_predator	C	6	-0.01661	0.000756	-21.961	2.205e-100	slope
Anc_prey	Evo_predator	A	7	-0.00594	0.000756	-7.849	5.499e-15	slope
Anc_prey	Evo_predator	B	8	-0.00906	0.000756	-11.980	1.879e-32	slope
Anc_prey	Evo_predator	C	9	-0.00704	0.000756	-9.308	2.211e-20	slope
Evo_prey	Evo_predator	A	10	0.00363	0.000756	4.798	1.672e-06	slope
Evo_prey	Evo_predator	B	11	0.01695	0.000756	22.408	3.564e-104	slope
Evo_prey	Evo_predator	C	12	0.01212	0.000756	16.022	7.565e-56	slope

Populations densities

Table S9a. Linear model predator densities. A generalised linear model (stats::lm in R, method = “Gaussian”, link = “identity”, equivalent to a normal linear model) with mean predator density as outcome variable was fit to determine the fixed effects of predator and prey evolutionary history. Formula: mean_predator_density ~ prey_history + predator_history + prey_history:predator_history. P-values < 0.05 were considered significant.

variable	Df	Deviance	Resid. Df	Resid. Dev	F	Pr(>F)
NULL			11	786,551,856.9644		
predator_history	1	350,522,034.7265	10	436,029,822.2379	39.9768	0.0002
prey_history	1	310,423,264.2229	9	125,606,558.0149	35.4036	0.0003
predator_history:prey_history	1	55,461,457.6622	8	70,145,100.3527	6.3253	0.0361

Table S9b. Analysis of deviance predator densities. Based on the linear model Table S7a an analysis of deviance (equivalent to an ANOVA) was performed (stats::anova).

	Estimate	Standard Error	z value	Pr(>|z|)	
(Intercept)	18,482.8000	1,709.5943	10.8112	0.0000	***
predator_historyevolved predator	15,108.9556	2,417.7314	6.2492	0.0002	***
prey_historyevolved prey	-5,872.5689	2,417.7314	-2.4290	0.0413	*
predator_historyevolved predator:prey_historyevolved prey	-8,599.3378	3,419.1885	-2.5150	0.0361	*

*Signif. codes: 0 <= '***' < 0.001 < '**' < 0.01 < '*' < 0.05*

(Dispersion parameter for gaussian family taken to be 8768138)

Null deviance: 7.866e+08 on 11 degrees of freedom

Residual deviance: 7.015e+07 on 8 degrees of freedom

(6 observations deleted due to missingness)

Table S9c. Pair-wise comparison of group means. Based on the ANOVA in Table S7b pairwise comparisons between all group means were performed (stats::TukeyHSD).

variable	diff	lwr	upr	p.adj
evolved predator:ancestral prey-naive predator:ancestral prey	15,108.9556	7,366.5286	22,851.3825	0.0011
naive predator:evolved prey-naive predator:ancestral prey	-5,872.5689	-13,614.9958	1,869.8580	0.1483
evolved predator:evolved prey-naive predator:ancestral prey	637.0489	-7,105.3780	8,379.4758	0.9931
naive predator:evolved prey-evolved predator:ancestral prey	-20,981.5244	-28,723.9514	-13,239.0975	0.0001
evolved predator:evolved prey-evolved predator:ancestral prey	-14,471.9067	-22,214.3336	-6,729.4797	0.0015
evolved predator:evolved prey-naive predator:evolved prey	6,509.6178	-1,232.8092	14,252.0447	0.1027

Table S10a. Linear model prey densities. A generalised linear model (stats::lm in R, method = "Gaussian", link = "identity", equivalent to a normal linear model) with mean prey OD as outcome variable was fit to determine the fixed effects of predator and prey evolutionary history. Formula: mean_prey_OD ~ prey_history + predator_history + prey_history:predator_history. P-values < 0.05 were considered significant.

	Estimate	Standard Error	z value	Pr(>|z|)	
(Intercept)	0.5599	0.0093	60.0851	0.0000	***
predator_historynaive predator	-0.3227	0.0132	-24.4843	0.0000	***
predator_historyevolved predator	-0.3154	0.0132	-23.9295	0.0000	***
prey_historyevolved prey	-0.0094	0.0132	-0.7150	0.4883	
predator_historynaive predator:prey_historyevolved prey	0.0647	0.0186	3.4721	0.0046	**
predator_historyevolved predator:prey_historyevolved prey	0.0410	0.0186	2.1987	0.0482	*

*Signif. codes: 0 <= '***' < 0.001 < '**' < 0.01 < '*' < 0.05*

(Dispersion parameter for gaussian family taken to be 0.0002605109)

Null deviance: 0.3518 on 17 degrees of freedom

Residual deviance: 0.003126 on 12 degrees of freedom

Table S10b. Analysis of deviance prey densities. Based on the linear model in Table S8a an analysis of deviance (equivalent to an ANOVA) was performed (stats::anova).

variable	Df	Deviance	Resid. Df	Resid. Dev	F	Pr(>F)
NULL			17	0.3518		
predator_history	2	0.3425	15	0.0093	657.3532	0.0000
prey_history	1	0.0030	14	0.0063	11.5047	0.0053
predator_history:prey_history	2	0.0032	12	0.0031	6.1705	0.0144

Table S10c. Pair-wise comparison of group means. Based on the ANOVA in Table S8b pairwise comparisons between all group means were performed (stats::TukeyHSD).

variable	diff	lwr	upr	p.adj
naive predator:ancestral prey-no predator:ancestral prey	-0.3227	-0.3669	-0.2784	0.0000
evolved predator:ancestral prey-no predator:ancestral prey	-0.3154	-0.3596	-0.2711	0.0000
no predator:evolved prey-no predator:ancestral prey	-0.0094	-0.0537	0.0348	0.9763
naive predator:evolved prey-no predator:ancestral prey	-0.2674	-0.3116	-0.2231	0.0000
evolved predator:evolved prey-no predator:ancestral prey	-0.2838	-0.3281	-0.2395	0.0000
evolved predator:ancestral prey-naive predator:ancestral prey	0.0073	-0.0370	0.0516	0.9923
no predator:evolved prey-naive predator:ancestral prey	0.3132	0.2690	0.3575	0.0000
naive predator:evolved prey-naive predator:ancestral prey	0.0553	0.0110	0.0996	0.0122
evolved predator:evolved prey-naive predator:ancestral prey	0.0389	-0.0054	0.0831	0.0983
no predator:evolved prey-evolved predator:ancestral prey	0.3059	0.2617	0.3502	0.0000
naive predator:evolved prey-evolved predator:ancestral prey	0.0480	0.0037	0.0922	0.0311
evolved predator:evolved prey-evolved predator:ancestral prey	0.0316	-0.0127	0.0758	0.2319
naive predator:evolved prey-no predator:evolved prey	-0.2580	-0.3022	-0.2137	0.0000
evolved predator:evolved prey-no predator:evolved prey	-0.2744	-0.3186	-0.2301	0.0000
evolved predator:evolved prey-naive predator:evolved prey	-0.0164	-0.0607	0.0278	0.8069

References

Auguie, Baptiste. (2017). *gridExtra: Miscellaneous Functions for "Grid" Graphics* (Version 2.3) [R]. https://CRAN.R-project.org/package=gridExtra

Benjamin, D., Sato, T., Cibulskis, K., Getz, G., Stewart, C., & Lichtenstein, L. (2019). *Calling Somatic SNVs and Indels with Mutect2*. https://doi.org/10.1101/861054

Bush, S. J. (2021). Generalizable characteristics of false-positive bacterial variant calls. *Microbial Genomics*, *7*(8). https://doi.org/10.1099/mgen.0.000615

Buskirk, S. W., Peace, R. E., & Lang, G. I. (2017). Hitchhiking and epistasis give rise to cohort dynamics in adapting populations. *Proceedings of the National Academy of Sciences*, *114*(31), 8330–8335. https://doi.org/10.1073/pnas.1702314114

Cairns, J., Moerman, F., Fronhofer, E. A., Altermatt, F., & Hiltunen, T. (2020). Evolution in interacting species alters predator life-history traits, behaviour and morphology in experimental microbial communities. *Proceedings of the Royal Society B: Biological Sciences*, *287*(1928), 20200652. https://doi.org/10.1098/rspb.2020.0652

Camargo, A. P., Roux, S., Schulz, F., Babinski, M., Xu, Y., Hu, B., Chain, P. S. G., Nayfach, S., & Kyrpides, N. C. (2023). Identification of mobile genetic elements with geNomad. *Nature Biotechnology*. https://doi.org/10.1038/s41587-023-01953-y

Cantalapiedra, C. P., Hernández-Plaza, A., Letunic, I., Bork, P., & Huerta-Cepas, J. (2021). eggNOG-mapper v2: Functional Annotation, Orthology Assignments, and Domain Prediction at the Metagenomic Scale. *Molecular Biology and Evolution*, *38*(12), 5825–5829. https://doi.org/10.1093/molbev/msab293

Cassidy-Hanley, D. M. (2012). Tetrahymena in the laboratory: Strain resources, methods for culture, maintenance, and storage. *Methods in Cell Biology*, *109*, 237–276. https://doi.org/10.1016/B978-0-12-385967-9.00008-6

Cingolani, P., Platts, A., Wang, L. L., Coon, M., Nguyen, T., Wang, L., Land, S. J., Lu, X., & Ruden, D. M. (2012). A program for annotating and predicting the effects of single nucleotide polymorphisms, SnpEff: SNPs in the genome of Drosophila melanogaster strain w ^{1118} ; iso-2; iso-3. *Fly*, *6*(2), 80–92.

https://doi.org/10.4161/fly.19695

Collins, K. (2012). *Tetrahymena thermophila* (1st ed). Elsevier/Academic Press.

Cooper, V. S. (2018). Experimental Evolution as a High-Throughput Screen for Genetic Adaptations. *mSphere*, *3*(3), e00121-18. https://doi.org/10.1128/mSphere.00121-18

De Cáceres, M., Coll, L., Legendre, P., Allen, R. B., Wiser, S. K., Fortin, M., Condit, R., & Hubbell, S. (2019). Trajectory analysis in community ecology. *Ecological Monographs*, *89*(2), e01350. https://doi.org/10.1002/ecm.1350

De Visser, J. A. G. M., & Rozen, D. E. (2006). Clonal Interference and the Periodic Selection of New Beneficial Mutations in *Escherichia coli*. *Genetics*, *172*(4), 2093–2100. https://doi.org/10.1534/genetics.105.052373

Dowle, Matt & Srinivasan, Arun. (2023). *data.table: Extension of "data.frame"* (Version 1.14.8) [R]. https://CRAN.R-project.org/package=data.table

Durrant, M. G., Li, M. M., Siranosian, B. A., Montgomery, S. B., & Bhatt, A. S. (2020). A Bioinformatic Analysis of Integrative Mobile Genetic Elements Highlights Their Role in Bacterial Adaptation. *Cell Host & Microbe*, *27*(1), 140-153.e9. https://doi.org/10.1016/j.chom.2019.10.022

Galperin, M. Y., Wolf, Y. I., Makarova, K. S., Vera Alvarez, R., Landsman, D., & Koonin, E. V. (2021). COG database update: Focus on microbial diversity, model organisms, and widespread pathogens. *Nucleic Acids Research*, *49*(D1), D274–D281. https://doi.org/10.1093/nar/gkaa1018

Gerrish, P. J., & Lenski, R. E. (1998). The fate of competing beneficial mutations in an asecual population. *Genetica*, *102/103*, 127–144. https://doi.org/10.1023/A:1017067816551

Good, B. H., McDonald, M. J., Barrick, J. E., Lenski, R. E., & Desai, M. M. (2017). The dynamics of molecular evolution over 60,000 generations. *Nature*, *551*(7678), 45–50. https://doi.org/10.1038/nature24287

Hogle, S. L., Hepolehto, I., Ruokolainen, L., Cairns, J., & Hiltunen, T. (2022). Effects of phenotypic variation on consumer coexistence and prey community structure. *Ecology Letters*, *25*(2), 307–319. https://doi.org/10.1111/ele.13924

Hogle, S. L., Tamminen, M., & Hiltunen, T. (2024). Complete genome sequences of 30 bacterial species from

a synthetic community. *Microbiology Resource Announcements*, e00111-24. https://doi.org/10.1128/mra.00111-24

Hothorn, T., Bretz, F., & Westfall, P. (2008). Simultaneous Inference in General Parametric Models. *Biometrical Journal*, *50*(3), 346–363. https://doi.org/10.1002/bimj.200810425

Huerta-Cepas, J., Szklarczyk, D., Heller, D., Hernández-Plaza, A., Forslund, S. K., Cook, H., Mende, D. R., Letunic, I., Rattei, T., Jensen, L. J., von Mering, C., & Bork, P. (2019). eggNOG 5.0: A hierarchical, functionally and phylogenetically annotated orthology resource based on 5090 organisms and 2502 viruses. *Nucleic Acids Research*, *47*(D1), D309–D314. https://doi.org/10.1093/nar/gky1085

King, T., Ishihama, A., Kori, A., & Ferenci, T. (2004). A Regulatory Trade-Off as a Source of Strain Variation in the Species *Escherichia coli*. *Journal of Bacteriology*, *186*(17), 5614–5620. https://doi.org/10.1128/JB.186.17.5614-5620.2004

Li, H. (2013). *Aligning sequence reads, clone sequences and assembly contigs with BWA-MEM* (Version 2). arXiv. https://doi.org/10.48550/ARXIV.1303.3997

Liu, X., & Ferenci, T. (1998). Regulation of Porin-Mediated Outer Membrane Permeability by Nutrient Limitation in *Escherichia coli*. *Journal of Bacteriology*, *180*(15), 3917–3922. https://doi.org/10.1128/JB.180.15.3917-3922.1998

Miller, K. A., Phillips, R. S., Mrazek, J., & Hoover, T. R. (2013). Salmonella Utilizes D-Glucosaminate via a Mannose Family Phosphotransferase System Permease and Associated Enzymes. *Journal of Bacteriology*, *195*(18), 4057–4066. https://doi.org/10.1128/JB.00290-13

Neuwirth, Erich. (2022). *RColorBrewer: ColorBrewer Palettes* (Version 1.1-3) [R]. https://CRAN.R-project.org/package=RColorBrewer

Oksanen, Jari, Simpson, Gavin L., Blanchet, F. Guillaume, Kindt, Roeland, Legendre, Pierre, Minchin, Peter R., O’Hara, R.B., Solymos, Peter, Stevens, M. Henry H., Szoecs, Eduard, Wagner, Helene, Barbour, Matt, Bedward, Michael, Bolker, Ben, Borcard, Daniel, Carvalho, Gustavo, Chirico, Michael, De Cacere, Miquel, Durand, Sebastien, … Weedon, James. (2022). *vegan: Community Ecology Package* (Version 2.6-4) [R]. https://CRAN.R-project.org/package=vegan

Pease, A. J., Roa, B. R., Luo, W., & Winkler, M. E. (2002). Positive Growth Rate-Dependent Regulation of the *pdxA* , *ksgA* , and *pdxB* Genes of *Escherichia coli* K-12. *Journal of Bacteriology*, *184*(5), 1359–1369. https://doi.org/10.1128/JB.184.5.1359-1369.2002

Pedersen, Thomans Lin. (2022). *patchwork: The composer of Plots* (Version 1.1.2) [R]. https://CRAN.R-project.org/package=patchwork

Saxer, G., Krepps, M. D., Merkley, E. D., Ansong, C., Deatherage Kaiser, B. L., Valovska, M.-T., Ristic, N., Yeh, P. T., Prakash, V. P., Leiser, O. P., Nakhleh, L., Gibbons, H. S., Kreuzer, H. W., & Shamoo, Y. (2014). Mutations in Global Regulators Lead to Metabolic Selection during Adaptation to Complex Environments. *PLoS Genetics*, *10*(12), e1004872. https://doi.org/10.1371/journal.pgen.1004872

Seemann, T. (2014). Prokka: Rapid prokaryotic genome annotation. *Bioinformatics*, *30*(14), 2068–2069. https://doi.org/10.1093/bioinformatics/btu153

Shoemaker, W. R., Polezhaeva, E., Givens, K. B., & Lennon, J. T. (2021). Molecular Evolutionary Dynamics of Energy Limited Microorganisms. *Molecular Biology and Evolution*, *38*(10), 4532–4545. https://doi.org/10.1093/molbev/msab195

Slowikowski. (2023). *ggrepel: Automatically Position Non-Overlapping Text Labels with "ggplot2"* (Version 0.9.4) [R]. https://CRAN.R-project.org/package=ggrepel

Sturbois, A., De Cáceres, M., Sánchez-Pinillos, M., Schaal, G., Gauthier, O., Mao, P. L., Ponsero, A., & Desroy, N. (2021). Extending community trajectory analysis: New metrics and representation. *Ecological Modelling*, *440*, 109400. https://doi.org/10.1016/j.ecolmodel.2020.109400

van den Brand, Teun. (2023). *ggh4x: Hacks for "ggplot2"* (Version 0.2.6) [R]. https://CRAN.R-project.org/package=ggh4x

van der Auwera, G., & O'Connor, B. D. (2020). *Genomics in the Cloud: Using Docker, GATK, and WDL in Terra*. O'Reilly Media, Incorporated. https://books.google.fi/books?id=wwiCswEACAAJ

Wichman, H. A., Badgett, M. R., Scott, L. A., Boulianne, C. M., & Bull, J. J. (1999). Different Trajectories of Parallel Evolution During Viral Adaptation. *Science*, *285*(5426), 422–424. https://doi.org/10.1126/science.285.5426.422

Wickham, H., Averick, M., Bryan, J., Chang, W., McGowan, L., François, R., Grolemund, G., Hayes, A., Henry, L., Hester, J., Kuhn, M., Pedersen, T., Miller, E., Bache, S., Müller, K., Ooms, J., Robinson, D., Seidel, D., Spinu, V., … Yutani, H. (2019). Welcome to the Tidyverse. *Journal of Open Source Software*, *4*(43), 1686. https://doi.org/10.21105/joss.01686
